# Cluster-randomized controlled trial of a mobile produce market designed to address diet and food insecurity in underserved communities

**DOI:** 10.1186/s40795-026-01302-7

**Published:** 2026-04-09

**Authors:** Lucia A. Leone, Christina Kasprzak, Anne Lally Mathiebe, Rocco Paluch, Lindsey Haynes-Maslow, Samina Raja, Laurene Tumiel-Berhalter, Leah N. Vermont, Alice Ammerman

**Affiliations:** 1 Department of Community Health and Health Behavior, School of Public Health and Health Professions, University at Buffalo, 333 Kimball Tower, Buffalo, NY 14214 USA; 2 Department of Anthropology, The College of Arts and Sciences, University at Buffalo, Buffalo, NY 14261 USA; 3 Department of Pediatrics, Jacobs School of Medicine and Biomedical Sciences, University at Buffalo, Conventus Building, Room 5419, Buffalo, NY 14203 USA; 4 Department of Health Policy and Management, University of North Carolina at Chapel Hill , 1105B McGavran-Greenberg Hall, CB #7411, Chapel Hill, NC 27599-7411 USA; 5 Department of Urban and Regional Planning, School of Architecture and Planning, University at Buffalo, Buffalo, NY 14214 USA; 6 Department of Family Medicine, Jacobs School of Medicine and Biomedical Sciences, University at Buffalo, 77 Goodell Street, Buffalo, NY 14203 USA; 7 Department of Nutrition, University of North Carolina at Chapel Hill, Center for Health Promotion and Disease Prevention, 1700 Martin Luther King Boulevard, CB #7426, Chapel Hill, NC 27599 USA

**Keywords:** Cluster-randomized trial, Food environment, Fruits and vegetables, Lower-income, Mobile market

## Abstract

**Background:**

Veggie Van (VV) is a multi-level mobile produce market intervention that increased fruit and vegetable (F&V) consumption in lower-income communities in an efficacy study. The aim of this study was to understand the impact of the VV intervention delivered by multiple community partners on food security and diet-related behaviors.

**Methods:**

This cluster-randomized trial was conducted in partnership with nine organizations (e.g., food focused non-profits) across four states in the United States. Partner organizations selected 33 community sites that reached individuals with lower-income and limited access to healthy food. Eligible study participants were age 18+, the primary household shopper, and lived near/frequented the community site; 699 participants were enrolled across all communities. Sites were randomized in pairs to receive the VV intervention (*n* = 17 sites) or a planning condition (*n* = 16 sites) for one year.

Outcomes were change in food security, F&V consumption measured by food frequency and 24-hour recalls, dermal carotenoid levels and nutrition-related psychosocial measures. Generalized linear mixed models were used to examine the effect of the intervention while adjusting for clustering within sites and baseline values. Additional analyses compared individuals based on their utilization of the VV (users/non-users) and communities that entered the study after peak COVID-19 shutdowns (Post-COVID sites).

**Results:**

In the intent-to-treat analyses, there were no statistically significant differences in food security or F&V consumption between intervention and control sites. VV users at post-COVID sites had significantly better (p=0.01) average food security scores (n=102, adj. mean=1.4, SE=0.18) after 1 year than non-users (n=201, adj. mean=2.0, SE 0.15).

**Conclusions:**

This research was the first study to evaluate the effectiveness of mobile markets across states, when implemented by different community organizations. Study implementation, recruitment, and data collection were significantly altered by the COVID-19 pandemic. While the magnitude of changes in F&V intake at post-COVID sites was similar to previous research, we did not find statistically significant differences between groups for F&V intake. Analyses also indicated that VV users improved their food security. Future research will examine how fidelity to the VV model by partners affected participant outcomes.

**Trial registration:**

This trial was registered at https://www.clinicaltrials.gov on January 29, 2020 (NCT04246593).

**Supplementary Information:**

The online version contains supplementary material available at 10.1186/s40795-026-01302-7.

## Background

In 2022, 44.2 million Americans (12.8%) lived in food-insecure households. The rate of food insecurity was substantially higher for Black (22.4%) and Hispanic (20.8%) households and those with incomes below 185% of the federal poverty level (32.0%) [1]. Beyond food security, nutrition security is defined as having consistent access, availability, and affordability to foods and beverages that promote well-being and prevent disease, such as fruits and vegetables (F&V) [2]. While nutrition security is a relatively new concept in the US and historical measurement is not available, measures of healthy diet, such as F&V consumption can serve as a proxy at the population-level [2]. In 2019, only 6.8% of individuals with lower-income reported meeting F&V recommendations [3] with most Americans needing to increase their total daily F&V intake by at least 1 cup to meet recommendations [4].

F&V consumption is a critical component of disease prevention. Adults who consume more produce are less likely to develop heart disease, diabetes, certain types of cancer, and are more likely to sustain a healthy weight [5–8]. Furthermore, multiple meta-analyses show a dose-response for eating more F&V on multiple disease outcomes [5, 9, 10]; notably, increasing intake from 1.5 servings/day (average American intake) to 3 servings/day could reduce all-cause mortality by 7% population-wide [9]. In the United States (US), substantial socioeconomic disparities exist in chronic disease prevalence, and poor diets among Americans with lower-income are a significant contributor [10–12]. Limited availability of high quality, affordable F&V, coupled with a greater prevalence of fast-food outlets in lower-income and minority communities, are partially responsible for poor diets among residents [13–15].

Policy has been enacted at the national, state, regional and local levels to increase healthy food retail in underserved communities to address disparities in access to healthy food. However, the research supporting these initiatives is limited. Early policies focused on opening new supermarkets in lower-income communities; however, a review of the literature found no impact of the introduction of new supermarkets on F&V intake among residents with lower-income [16]. One landmark study of a new grocery store in an underserved neighborhood found an overall improvement in diet quality and perceived access to healthy food, but these changes occurred regardless of whether residents were frequent shoppers of the grocery store [17]. One reason that impacts on diet may have been limited is that building new retail stores addresses food availability, but not other key dimensions of food access (i.e., affordability, acceptability and accommodation) [16, 18]. While affordability is a commonly cited predictor of F&V consumption, quality is also a high priority for consumers with lower-income [19, 20]. A study in lower-income communities in Chicago, IL found a positive association between diet and perceptions of the food environment; those who rated their food shopping options as higher in quality, selection, and convenience had greater F&V consumption [21].

Mobile markets, often referred to as a “farmers’ market on wheels” bring a variety of high quality affordable fresh fruits, vegetables, and healthy foods to convenient locations in underserved communities [22]. Mobile markets are not only acceptable, affordable food option in lower-income communities [20, 23, 24], but they have been shown to increase F&V consumption in several studies [25–28]. Compared to other new food retail options (i.e., supermarkets or farmers markets), mobile markets more completely address the multidimensional aspects of food access [16]. While there are different types of mobile market models, the Veggie Van (VV) model was created based on multiple research studies [23, 27] including a previous cluster-randomized controlled trial (RCT) in 12 communities in North Carolina [29]. In that trial, the VV intervention increased F&V intake by ≈ 1 cup/day (about 2 servings) among adults [26]. These dietary improvements are remarkable considering behavioral interventions in lower-income populations generally show improvements in the 0.6 to 0.95 servings/day range [30, 31].

Despite promising results for the VV program and similar mobile market programs [27, 28, 32–34] previous studies are limited by several factors. First, they were evaluating mobile markets operated by only a single organization. For example, in the previous VV study, while a randomized controlled trial, the intervention was implemented by just one organization across 12 communities located within a small, mainly urban area. Further, most of these studies relied on food frequency questionnaires or screeners rather than the gold standard 24-hour recall for dietary data collection [35]. The current study fills a gap in mobile market research by studying the effectiveness of the VV intervention when delivered by multiple organizations. Further, the current study used gold standard and objective dietary data collection including 24-hour recalls and dermal carotenoids to estimate F&V consumption. In addition, the current study looked at food security, which had not been examined in previous RCTs of mobile markets but is a common focus for mobile market operators. This paper presents the outcomes from a cluster-randomized controlled trial delivered in 33 communities by partner organizations across four states evaluating the VV’s impact on F&V consumption, food security, food access and diet-related constructs. The study team hypothesized that the introduction of mobile markets following the VV model in new communities would alter the food environment, improve self-efficacy for eating fruits and vegetables, food security, and increase F&V intake among participants. Furthermore, that participants who shopped at the VV would improve their F&V intake and food security more than those who did not use the VV.

## Methods

This cluster-randomized trial was conducted in 33 communities across four states (New York, North Carolina, Georgia and Ohio); baseline data collection started at the first site in January 2020 and follow-up data collection ended at the last site in October 2023. Methods for this study have been previously reported [36, 37]. Briefly, we selected partner organizations (e.g., food-focused nonprofits) to run a VV intervention. Partners in turn selected community sites which were randomized to a VV intervention or delayed intervention control group. Partners and community sites worked together to identify participants interested in the study who were then recruited by the research team to participate in a 12-month study.

### Veggie Van intervention

VV is a multi-level evidence-based intervention intended for mobile produce markets [26, 29] Partners were supported by the research team to start a VV intervention at community sites that were randomized to the intervention condition. VV was previously tested in pilot and efficacy studies and found to have a favorable impact on F&V consumption in lower-income communities [25, 26]. The VV intervention is comprised of six core components designed to address the 5 A’s of food access (availability, affordability, accessibility, acceptability and accommodation) [38]. Components include: (1) regularly operating a mobile market in partnership with community sites located in convenient locations that are already serving lower-income communities and could promote the market to them, (2) offering a variety of fresh, high-quality F&V, (3) operating a reduced cost payment model, (4) accepting federal supplemental nutrition assistance program (SNAP) benefits and other available regional incentive programs, (5) offering regular cooking and nutrition education, and (6) offering and incentivizing customers to purchase a bundle of produce (multiple items for a set price) rather than just one or two items separately. Partners for this study were asked to run the VV intervention at community sites following these principles: run VV at least once per week at intervention sites for at least 10 months out of the 12-month study period, promote their market to potential customers in collaboration with the community site, and refrain from starting any new nutrition or food programs at the site during the intervention period. Partner organizations chose community sites primarily based on community need, but their interest and ability to sustain the mobile market site also needed to be demonstrated during the partner selection process. However, partners had ultimate discretion over whether they continued to operate the mobile market at participating VV study sites after the study period concluded.

### Delayed-intervention control condition

Partners were asked to coordinate a community engagement and planning process at community sites that were randomized to the delayed-intervention control condition. The goal of this process was to determine over the 12-month study period if the VV was a good fit for the community site and surrounding community or if a different food access program would be more appropriate. This planning and engagement period also facilitated the recruitment of study participants. Representatives from community sites in the delayed-intervention control condition were asked to refrain from starting any new nutrition or food programs at the site during the study period. After the 12-month study period, partners and community sites could jointly decide whether to launch a mobile market based on feedback received from the community during the planning period.

### Partner and site selection and randomization

Nine community-based organizations (e.g., food-focused nonprofits, cooperative extension) were selected to serve as partners for this research and implement the Veggie Van in their communities. The methods for selecting these partners and communities are described elsewhere [36]. Briefly, partners were selected through a “request for partners” process; each partner in turn identified 2–6 sites (based on partners’ capacity) in their region to potentially host a mobile market to be run by the partner. While initially nine partners were selected, one became non-responsive and was removed from the study; two additional partners were recruited over 2021–2022 to ensure there were enough participants to maintain study power.

Partners chose community sites that were randomized to either host a mobile market or serve as a delayed-intervention control site engaged in planning activities for the intervention period. These community sites were chosen because they had a history of serving lower-income and or food insecure communities but had not previously hosted a mobile market. Selected sites included libraries, community centers, senior centers, adult education programs, community schools, housing authorities, federally qualified healthcare centers, neighborhood associations, parks, etc.

After confirming participation in the study, pairs of community sites were randomized with each site in a pair being randomized to either the intervention condition or the delayed-intervention control condition. After randomization, the study team, partners, and community sites signed a memorandum of understanding (MOU) and established a study timeline for each site.

### Participant recruitment and retention

Participant recruitment, enrollment and data collection are described in detail elsewhere [37].

Briefly, partners facilitated participant recruitment for the study primarily through a ≈ 2-month community engagement period prior to the mobile market launch at intervention sites, or initiation of planning activities at delayed-intervention control sites. Partners and community sites at both intervention and control sites used interest forms to identify participants who expressed that they would be likely to use a mobile market; however, participants were not given a definitive mobile market launch date. If recruitment goals were not met within this period, study recruitment continued for up to two months after the mobile market was launched (intervention sites) or commencement of planning activities (delayed-intervention control sites). To be eligible for the study, individuals had to be at least 18 years old, able to speak English and/or Spanish, be the primary grocery shopper for their household, and self-report that they lived closed by or usually visited the site at least once a week, Individuals were ineligible if they were planning to leave the area or stop frequenting the site within the next year. To promote retention, all enrolled participants received quarterly newsletters via mail and or/e-mail (based on participant preference) from the research team with reminders about upcoming data collection, general updates about the study and its importance, and seasonal recipes.

### Data collection

Baseline data collection for each site pair took place prior to or within two months of the launch of the mobile market or planning activities. Follow-up data collection took place approximately 10–12 months post-baseline while the mobile market was still operating at the intervention site, and before the delayed-intervention control site launched a mobile market or other food access intervention. Data were collected at each time point using three methods: (1) phone surveys, (2) 24-hr dietary recalls, and (3) in-person data collection events to collect height/weight and Veggie Meter readings. Phone surveys lasted approximately 30 min and collected demographic data, food frequencies, food security, psychosocial measures and shopping relate behaviors. Refer to supplementary materials 4–5 for the baseline and follow-up surveys.

Twenty-four-hour recalls were administered over the phone using Nutrition Data System for Research (NDSR) software [39, 40]. Dietary recalls were conducted by a trained research assistant using a multi-pass interview approach. During the recall, participants referred to a portion size booklet that was mailed to each participant in advance of the first 24-hour recall and confirmed to be present at the time of the recall. Using the NDSR software, the research assistant guided participants through a recall of foods consumed the day prior for both a weekday and weekend to account for any dietary variability across the week at both baseline and follow-up. Participants who were enrolled in the study after the mobile market launched at intervention sites did not complete a 24-hr dietary recall due to the possibility of their consumption data being influenced by already shopping at the market.

In-person data collection events were hosted jointly between the partner, study team, and community site at baseline and follow-up. The study team also trained partner staff on data collection procedures to enable them to complete any remaining data collection activities with participants who were unable to attend the events. Due to disruptions caused by the COVID-19 pandemic, not all sites were able to host in-person data collection events. Participants could earn up to $45 at each time point (baseline and follow-up) for completing a survey, two-24-hour dietary recalls, and the in-person data collection for a total of up to $90.

### Measures

#### Demographics

Gender was self-reported using an open-ended survey question and subsequently coded in analysis. Racial and ethnic identification were also self-reported using the convention established by the 2020 United States Census. Additional demographics included age, household income, marital status, education, employment status, government assistance participation in the past 12 months (e.g., SNAP, Special Supplemental Nutrition Assistance Program for Women, Infants, and Children [WIC], Medicaid, Temporary Assistance for Needy Families [TANF], Head Start, etc.), total mouths to feed within the household (children and adults), and length of residency at their current address.

#### Dietary and anthropometric measures

The main outcome was change in F&V intake between baseline and 12-month follow-up. Additional details on these measures are described elsewhere [37]. Briefly, the survey assessed intake (time/day) using the 2017 Behavioral Risk Factor Surveillance System (BRFSS) Fruit and Vegetable module [41]. Average F&V intake (servings/day) was also calculated using two 24-hour recalls (one weekend and one weekday). The F&V variable from NDSR included all fruits, juices and vegetables consumed. Dermal carotenoids were measured using the Veggie Meter™, a finger scan device that relies on pressure mediated Raman Spectroscopy (RS) as a non-invasive and valid indicator of changes in skin carotenoids in response to dietary carotenoid consumption [42, 43]. Lastly, height and weight measurements were collected via phone survey and in-person data collection events (when possible) at baseline and 12 months and used to calculate body mass index (BMI). However, due to COVID-related cancellations of in-person data collection events, in-person height and weight were collected at a much lower rate compared to self-reported height and weight collected via phone survey. Therefore, self-reported height and weight were used to calculate BMI and for descriptive analyses of the sample. Due to cancellations, dermal carotenoid data was also not collected from some participants at baseline and/or follow-up.

#### Food security

Food security status in the past 12 months was measured using the United States Department of Agriculture (USDA) 10-item US Adult Food Security Survey; [44] resulting raw scores were analyzed as a continuous outcome. In addition, raw scores were also converted to one of four categories of food security defined by the USDA (high food security [0], marginal food security [1–2], low food security [3–5], or very low food security [6–10]) to analyze food security as a categorical variable [44]. A subgroup of food insecure participants, including those with a score of 3 or higher (low and very low food security), was created to conduct exploratory analyses.

#### Psychosocial measures

To assess the impact of the intervention on the food environment, perceptions of access to fresh F&V were evaluated using a 3-item scale derived from the Perceived Nutrition Environment Measures Survey (NEMS-P) [45]. This scale assesses price, quality, and availability of F&V within one-mile or a 30-minute walk of a participant’s home [46] and had been previously adapted to also measure access near the community site and in general [29]. Perceived access scores range from 3 (strongly agree with all items) to 15 (strongly disagree with all items), with a midpoint of 8 indicating a neutral response. Responses were reverse coded prior to analysis to aid in interpretation so that a higher score indicated greater perceived access.

The impact of the nutrition education component of the VV intervention was assessed by evaluating changes in self-efficacy and perceived behaviors. Self-efficacy to purchase, prepare, and eat F&V was assessed using a selection of items adapted from a study of shoppers where self-efficacy was shown to be correlated with nutrition behaviors [47]; this measure includes a 10-point Likert scale with responses ranging from 1 to 10 (1 = “very easy” to 10 = “very hard”). Responses were reverse coded prior to analysis to aid in interpretation so that a higher score indicates greater self-efficacy. Items were also summed to create a total self-efficacy score (range 8 to 80). Perceived barriers to eating F&V were measured using twelve questions with response options ranging from 1 (strongly disagree) to 4 (strongly agree) with a higher score indicating greater perceived barriers (range 4 to 48); this measure has been previously tested in adults with lower-income [48] and reflects common barriers found in the literature [19, 49–51].

### Analysis

All analyses were completed using SAS 9.4. To evaluate the impact of the VV intervention on change in F&V intake using intent-to-treat principles (i.e., categorizing participants based on site randomization status). For continuous outcomes, PROC GLIMMIX was used to conduct generalized linear mixed models (GLMM) with a random intercept to control for clustering within sites. Change scores were calculated for all variables to assess the difference between 12-month follow-up and baseline, by subtracting baseline values from 12-month follow-up values. Difference-in-difference analyses were conducted to assess the mean difference of change scores, or the intervention effect, between the intervention and control groups. Cohen’s *d* was calculated to quantify the effect size for each outcome. To further explore the intervention effect, GLMMs were fit to adjust for [1] baseline dietary intake and [2] race and baseline income due to statistically significant differences between in these variables between intervention and control groups at baseline (race *p*=0.02; income: *p* = 0.02). A sensitivity analysis excluding extreme F&V reporters (BRFSS: *n* = 2; NDSR: *n* = 3), defined as participants who had a change (increase or decrease) greater than 10 servings/times of F&V per day, was also conducted. This threshold was informed by the past VV efficacy study [52] and further justified by generating histograms to identify outliers in the data distribution for the current study.

In addition to intent-to-treat analyses, additional planned analyses compared change in F&V intake for those who reported ever shopping at VV at their community site (VV users) to those who did not report shopping at VV (VV non-users), including delayed-intervention control participants. Post-hoc analyses examined differences among a sub-sample of sites (*n* = 17 sites) that launched in 2021 or later after the peak of COVID-19 related closures (i.e., post-COVID sites). Secondary outcome analyses were conducted using GLMMs and controlled for baseline covariates of interest and clustering within sites. Exploratory Chi-square analyses examined improvement in food security as a dichotomous variable among the sub-group of participants who were food insecure at baseline (score of 4–10 on the food security module).

## Results

### Recruitment, enrollment and retention

**Fig. 1:**
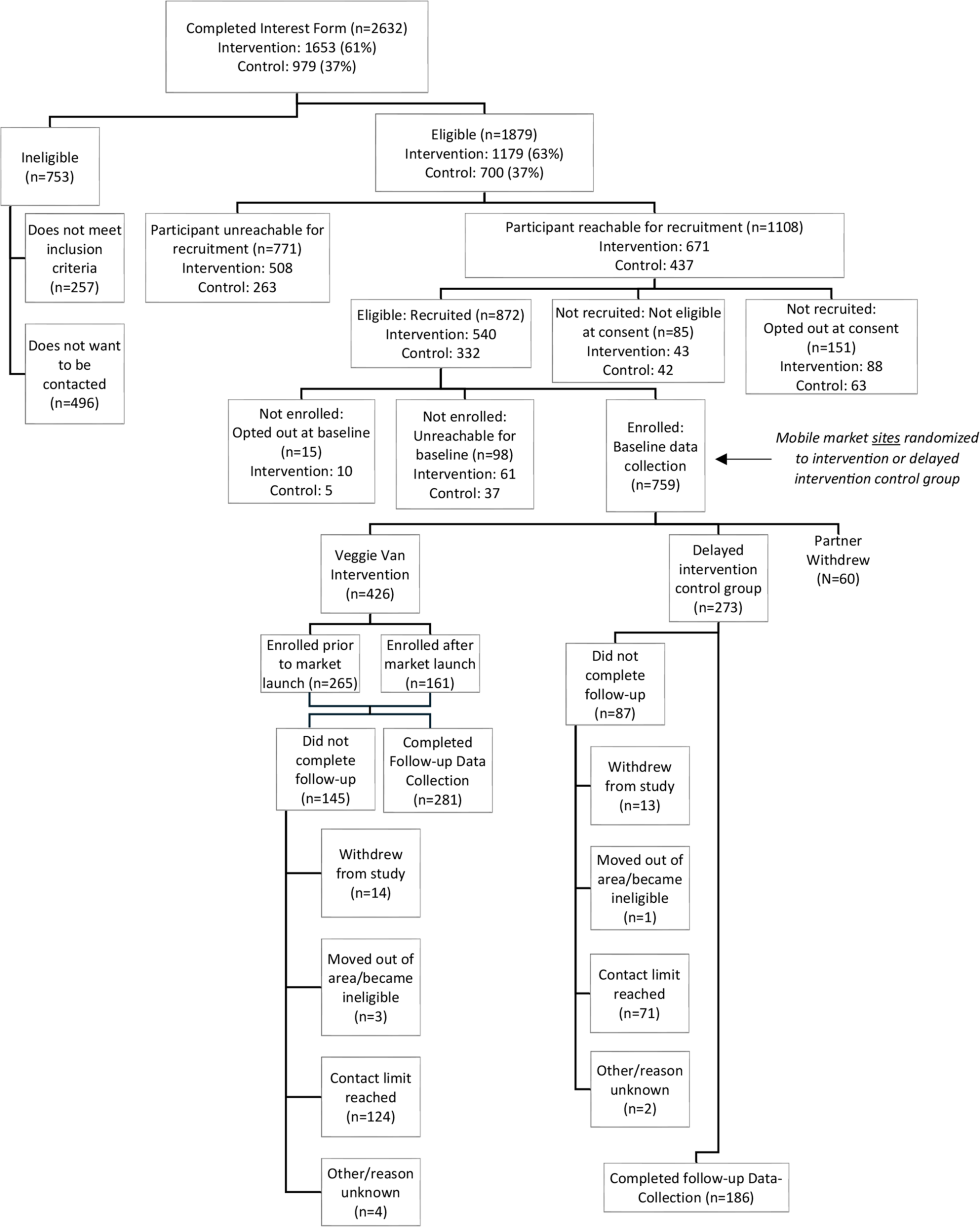
Flow diagram of participant recruitment, eligibility, enrollment, and retention in a randomized controlled trial of a mobile produce market intervention

Figure 1 outlines study recruitment, enrollment, and retention. Interest forms from 2,632 individuals were collected across all community sites. Of the interest forms received, 1,879 people were eligible, 1,108 were reachable for recruitment and 872 were eligible based on the recruitment phone call. Baseline surveys and study enrollment were completed with 759 individuals; however, the baseline participant data (*n* = 60) was removed for one partner that shut-down due to COVID-19 and had to withdraw from the study, leaving a final baseline sample size of 699 (426 intervention and 273 control participants). Follow-up data collection was completed with 467 people: 281 intervention participants and 186 control participants. There were no differences in attrition between the two groups; there was 64% completion among intervention participants compared to 61% among control participants (Chi-Square = 0.52, *p* = 0.47).

### Participant characteristics

Baseline characteristics for participants, by treatment arm, are presented in Table 1. Overall, the sample was predominantly female (84.3%), Black or African American (46.4%), and not married or living with a partner (65%). On average, the sample was 48.2 (SD 15.0) years old, had a mean BMI of 31.1 (SD 7.8) and had 2 adults and 1 child living in the household. More than half (60.5%) made less than $40,000 per year and received some form of government assistance (e.g., Medicaid, SNAP) in the past 12 months (65.2%). Additionally, 34.6% of the sample was food insecure at baseline.

**Table 1:** Veggie Van study participant baseline characteristics by intervention group (*N* = 699)

Variable	Intervention *n* = 426	Control *n* = 273	Entire Sample *n* = 699
Age, mean ± SD	47.1 ± 14.5	50.1 ± 15.5	48.2 ± 15
18–24 years, n (%)	14 (3.4)	8 (3.1)	22 (3.3)
25 to 34 years, n (%)	87 (21.3)	46 (18.0)	133 (20.0)
35 to 44 years, n (%)	86 (21.0)	46 (18.0)	132 (19.8)
45 to 64 years, n (%)	172 (42.1)	97 (37.9)	269 (40.5)
65 to 84 years, n (%)	48 (11.7)	57 (22.3)	105 (15.8)
85 to 99 years, n (%)	1 (0.2)	2 (0.8)	3 (0.5)
100 years and older, n (%)	1 (0.2)	0 (0)	1 (0.2)
BMI, ^a^ mean ± SD	30.6 ± 7.5	31.9 ± 8.3	31.1 ± 7.8
Adults to Feed in Household, mean ± SD	1.8 ± 0.8	1.8 ± 0.8	1.8 ± 0.8
Children to Feed in Household, mean ± SD	1.1 ± 1.1	0.9 ± 1.2	1 ± 1.3
Household Income, mean ± SD	33,686.9 ± 18,491.7	30,791.5 ± 19,411.2	32,542.0 ± 18,899.0
Food Insecure,^b^ n (%)	145 (34.2)	96 (35.3)	241 (34.6)
Gender, n (%)			
Male	64 (15.0)	43 (15.7)	107 (15.3)
Female	365 (84.3)	230 (84.3)	589 (84.3)
Other	2 (0.005)	0 (0)	3 (0.4)
Missing	1(0.002)	0(0)	0(0)
Marital Status, n (%)			
Married or living with a partner	157 (36.9)	84 (31.1)	241 (34.7)
Single	188 (44.1)	129 (47.8)	317 (45.6)
Divorced	44 (10.3)	32 (11.7)	76 (10.9)
Separated/Widowed	36 (8.5)	25 (9.2)	61 (8.7)
Missing	1 (0.2)	3 (1.1)	4 (0.5)
Education, n (%)			
Less than Grade 8	9 (2.1)	4 (1.5)	13 (1.9)
Some high school/high school grad/GED ^c^	103 (24.2)	69 (25.3)	172 (24.6)
Trade or beauty school graduate	24 (5.6)	14 (5.1)	38 (5.4)
Some college	97 (22.8)	73 (26.7)	170 (24.3)
College graduate	120 (28.2)	78 (28.6)	198 (28.3)
Postgraduate	71 (16.7)	34 (12.5)	105 (15.0)
Missing	2 (0.5)	1 (0.4)	3 (0.4)
Annual Household Income, n (%)			
Less than $10,000	59 (13.9)	63 (23.1)	122 (17.5)
$10,000 to $19,999	69 (16.2)	51 (18.7)	120 (17.2)
$20,000 to $29,999	64 (15.0)	25 (9.2)	90 (12.9)
$30,000 to $39,999	54 (12.7)	36 (13.2)	90 (12.9)
$40,000 to $49,999	40 (3.4)	17 (6.2)	57 (8.2)
$50,000 to $59,999	35 (8.2)	18 (6.6)	53 (7.6)
$60,000 or more	75 (17.6)	49 (18.0)	124 (17.7)
Missing	30 (7.0)	67 (25.9)	44 (6.3)
Employment Status, n (%)			
Employed full-time	101 (23.7)	63 (23.1)	164 (23.5)
Employed part-time	34 (8.0)	8 (2.9)	42 (6.0)
Unemployed	53 (12.4)	25 (9.2)	78 (11.2)
Retired	27 (6.3)	30 (11.0)	57 (8.2)
SSI/Disability ^d^	25 (5.9)	17 (6.2)	42 (6.0)
Other Work	2 (0.5)	1 (0.4)	3 (0.4)
Missing	184 (43.2)	129 (47.3)	313 (44.8)
Ethnicity, n (%)			
Not Hispanic or Latino/Latina	374 (87.8)	252 (92.3)	626 (89.6)
Hispanic or Latino/Latina	48 (11.3)	21 (7.7)	69 (9.9)
Missing	4 (0.6)	0 (0)	4 (0.6)
Race, n (%)			
American Indian/Native American	5 (1.2)	1 (0.4)	6 (0.9)
Asian	9 (2.1)	5 (1.8)	14 (2.0)
Black or African American	174 (40.9)	150 (55.0)	324 (46.4)
Multiracial	17 (4.0)	10 (3.7)	27 (3.9)
Native Hawaiian/Pacific Islander	2 (0.50)	0 (0)	2 (0.30)
White	189 (44.4)	92 (33.7)	281 (40.2)
Missing	30 (7.0)	15 (5.5)	45 (6.4)
Any Government Assistance,^e^ n (%)			
Yes	274 (64.3)	182 (66.7)	456 (65.2)
No	148 (34.7)	86 (31.5)	234 (33.5)
Missing	4 (0.9)	5 (1.8)	9 (1.3)
Food Assistance: SNAP,^f^ WIC,^g^ and/or Food Pantry, n (%)			
Yes	211 (49.5)	140 (51.3)	351 (50.2)
No	211 (49.5)	128 (46.9)	339 (48.5)
Missing	4 (0.90)	5 (1.8)	9 (1.3)
Social Services Benefits in the past 12 months, n (%)			
SNAP	160 (37.6)	112 (41.0)	272 (38.9)
WIC	54 (12.7)	17 (6.2)	71 (10.2)
Medicaid	176 (41.3)	110 (40.3)	286 (40.9)
Food Pantry	105 (24.7)	80 (29.3)	185 (26.5)
Free or reduced-price school breakfast/lunch	112 (26.3)	63 (23.1)	175 (25.0)
Social Security Disability Benefits	91 (21.4)	87 (31.9)	178 (25.5)
Headstart	26 (6.1)	8 (2.9)	34 (4.9)
TANF ^h^	19 (4.5)	10 (3.7)	29 (4.2)
None	135 (31.7)	76 (27.8)	211 (30.2)
Missing	4 (0.9)	5 (1.8)	9 (1.3)

^a^ BMI = Body-mass-index

^b^ Food insecure is defined as low or very low food security or a food security score of 3–10

^c^ GED = General Education Development

^d^ SSI = Social Security Income

^e^ Government assistance includes participation in any of the following programs: Supplemental Nutrition Assistance Program, Special Supplemental Nutrition Program for Women, Infants, and Children, Medicaid, Free or reduced-price school breakfast or lunch, Social Security Disability Benefits, Head Start, or Temporary Assistance for Needy Families

^f^ SNAP = Supplemental Nutrition Assistance Program

^g^ WIC = Special Supplemental Nutrition Program for Women, Infants, and Children

^h^ TANF = Temporary Assistance for Needy Families

Among all participants, 171 participants (40.1% of intervention arm) reported using the VV in the past year (VV users) and 369 did not (VV non-users); there were significant differences between VV users and non-users for income, employment status, and race and ethnicity. A total of 406 participants (intervention: *n* = 247; control: *n* = 161) were from sites that entered the study in 2021 or later (post-COVID sites). Within the COVID versus post-COVID sub-sample, there were significant differences between groups for income, employment status, race and ethnicity, and participation in government and food assistance programs.

### Fruit and vegetable intake

#### Fruit and vegetable intake for intervention vs. control

Dietary intake data are presented in Table 2. A total of 380 participants were recruited prior to market launch and completed at least one dietary recall at baseline; 290 participants completed dietary recalls at follow-up. However, only 212 individuals completed a dietary recall at both time points. Baseline F&V mean intake, measured through 24-hour dietary recall, was 4.8 (0.4) servings/day for the intervention group and 4.2 (0.4) servings/day for the control group (Range for study sample at baseline: 0-45.7 servings/day). At follow-up, the mean difference of -0.8 (0.7) servings/day between groups was not significant (*p* = 0.27). The differences remained non-significant after controlling for baseline F&V intake, income, and race, removing participants with extreme intake, and limiting the sample to post-COVID sites.

For F&V intake measured through BRFSS Survey Data, baseline intake was 3.3 (0.1) times/day for the intervention group and 3.1 (0.1) times/day for the control group (Range for study sample at baseline: 0-26.5 times/day). At follow-up, the mean difference was not significant (*p* = 0.30). This pattern of association and non-significance remained when controlling for baseline covariates, removing extreme values, and conducting sensitivity analyses among post-COVID sites.

**Table 2:** Impact of the Veggie Van on Participants’ Fruit and Vegetable Intake in the Veggie Van Study

Total Fruit and Vegetable Intake (Servings/Day) from 24-Hour Recall (NDSR ^a^)					
Outcome	Intervention (*n* = 426)	Control (*n* = 273)	Intervention Effect	*p*-value	*n*
	Mean (SE)	Mean (SE)	Mean Difference (SE)		
*Baseline* ^b^	4.8 (0.4)	4.2 (0.4)	0.5 (0.5)	0.34	380
*12-month Follow-up* ^b^	4.9 (0.4)	4.6 (0.4)	0.2 (0.6)	0.68	290
Change at 12-months ^b^					
	-0.5 (0.5)	0.3 (0.5)	-0.8 (0.7)	0.27	212
Change at 12-months controlling for baseline covariates (F&V intake, income, and race) ^b^					
	-0.4 (0.4)	0.2 (0.4)	-0.6 (0.5)	0.24	188
Change at 12-months controlling for baseline covariates (F&V intake, income, and race); extremes removed ^b^					
	-0.4 (0.3)	0.2 (0.3)	-0.6 (0.5)	0.25	186
Change at 12-months controlling for baseline covariates (F&V intake, income, and race); extremes removed; post-COVID ^c^ sites only ^b^					
	0.1 (0.4)	-0.4 (0.4)	0.5 (0.6)	0.43	101

Total Fruit and Vegetable Intake (Times/Day) from Survey Data (BRFSS ^d^)					
Outcome	Intervention	Control	Intervention Effect	*p*-value	*n*
	Mean (SE)	Mean (SE)	Mean Difference (SE)		
*Baseline* ^b^	3.3 (0.1)	3.1 (0.1)	0.2 (0.2)	0.27	684
*12-month Follow-up* ^b^	3.0 (0.1)	3.0 (0.2)	-0.02 (0.2)	0.90	446
Change at 12-months ^b^					
	-0.3 (0.1)	-0.09 (0.2)	-0.2 (0.2)	0.30	438
Change at 12-months controlling for baseline F&V intake, income, and race ^b^					
	-0.2 (0.1)	-0.2 (0.1)	0.02 (0.2)	0.91	379
Change at 12-months controlling for baseline covariates (F&V intake, income, and race); extremes removed ^b^					
	-0.2 (0.1)	-0.2 (0.1)	-0.002 (0.2)	0.99	377
Change at 12-months controlling for baseline covariates (F&V intake, income, and race); extremes removed; post-COVID ^c^ sites only ^b^					
	-0.1 (0.2)	-0.2 (0.2)	0.2 (0.2)	0.49	215

^a^ NDSR = Nutrition Data System for Research

^b^ GLMM = generalized linear mixed model; GLMM model was adjusted for clustering within sites

^c^ COVID = Coronavirus disease

^d^ BRFSS = Behavioral Risk Factor Surveillance System

#### Fruit and vegetable intake by Veggie Van usage

For F&V intake measured through dietary recall, when controlling for baseline covariates, the mean difference at follow-up between VV users and non-users was not statistically significant (*p* = 0.84). Among post-COVID sites, there was a 0.2 servings/day (0.5) increase in mean consumption among VV users compared to a reduction of 0.4 servings/day (0.3) in VV non-users, resulting in a non-significant mean difference of 0.6 (0.6) servings/day (*p* = 0.30; Cohen’s *d* = 0.3). There were small reductions (range: 0.1 to 0.3 times/day) in mean consumption for both groups (users and non-users) for BRFSS survey data when controlling for baseline covariates, removing extreme values, and looking at post-COVID sites only; however, no mean differences reached statistical significance.

#### Carotenoid intake

Carotenoid score data are presented in Table 3. A total of 217 people completed a Veggie Meter reading at baseline and 151 completed it at follow-up, but only 75 participants completed them at both time points. With a maximum possible score of 800, mean baseline carotenoid scores were 266.4 and 268.4 for the intervention and delayed-intervention control groups, respectively. At follow-up, mean carotenoid score for the intervention group decreased by 22.2 (29.9) while the delayed-intervention control group increased by 5.8 (31.3); though, the mean difference of -28 (43.3) was not statistically significant (*p* = 0.53; Cohen’s *d*: 0.3). The differences remained non-significant after controlling for baseline F&V intake, income, and race, and limiting the sample to post-COVID sites. Additional analyses between VV users and VV non-users failed to produce any significant mean differences between groups.

**Table 3:** Impact of the Veggie Van on participants’ dermal carotenoids in the Veggie Van study

Dermal Carotenoid Score ^a^ Measured by the Veggie Meter					
Outcome	Intervention (*n* = 426)	Control (*n* = 273)	Intervention Effect	*p*-value	*n*
	Mean (SE)	Mean (SE)	Mean Difference (SE)		
*Baseline* ^b^	266.4 (22.5)	268.4 (24.4)	-2.0 (33.2)	0.95	217
*12-month Follow-up* ^b^	242.3 (16.0)	275.7 (17.0)	-33.4 (23.4)	0.17	151
Change at 12-months ^b^					
	-22.2 (29.9)	5.8 (31.4)	-28.0 (43.3)	0.53	75
Change at 12-months controlling for baseline covariates (carotenoid score, income, and race) ^b^					
	-22.4 (19.8)	9.2 (20.0)	-31.5 (28.6)	0.30	61
Change at 12-months controlling for baseline covariates (carotenoid score, income, and race); post-COVID ^c^ sites only ^b^					
	-21.0 (19.0)	-1.8 (19.5)	-19.2 (27.7)	0.50	60

^a^Dermal carotenoid score is an indicator of consumption of carotenoid-rich fruits and vegetables. Dermal carotenoid score, as measured by the Veggie Meter, can range from a minimum of 0 to a maximum of 800

^b^GLMM – generalized linear mixed model; GLMM model was adjusted for clustering within sites

^c^COVID = Coronavirus disease

### Food Security

Changes in food security, measured on a continuous scale (0–10), are reported in Table 4. Although there were reductions in mean food security scores in the intervention group, there were no statistically significant changes between groups at follow-up (Baseline food security range: 0–10, median:1.0; follow-up range: 0–10, median: 0). When looking at post-COVID sites, intervention participants experienced a decrease in mean food security score while control participants experienced an increase; however, the mean difference of -0.5 (0.3) between groups was not statistically significant (*p* = 0.06; Cohen’s *d* = 0.3).

**Table 4:** Impact of the Veggie Van on participants’ food security status in the Veggie Van study

Food Security ^a^					
Outcome	Intervention (*n* = 426) Mean (SE)	Control (*n* = 273) Mean (SE)	Mean Difference (SE)	*p*-value	*n*
*Baseline* ^b^	2.3 (0.2)	2.1 (0.3)	0.2 (0.3)	0.60	696
*12-Month Follow-up* ^b^	1.6 (0.2)	1.8 (0.2)	-0.2 (0.3)	0.58	465
Change at 12-months ^a^					
	-0.4 (0.2)	-0.2 (0.2)	-0.2 (0.2)	0.47	464
Change at 12-months controlling for baseline covariates (food security, income, and race) ^b^					
	-0.4 (0.2)	-0.2 (0.2)	-0.2 (0.2)	0.44	401
Change at 12-months controlling for baseline covariates (food security, income, and race); post-COVID ^c^ sites only ^b^					
	-0.3 (0.2)	0.2 (0.2)	-0.5 (0.3)	0.06	228

^a^Food security score ranges from 0 to 10 with a lower score indicating high food security and a higher security indicating lower food security (food insecurity)

^b^GLMM – generalized linear mixed model; GLMM model was adjusted for clustering within sites

^c^COVID = Coronavirus disease

Table 5 shows the relationship between reported usage of VV and food security. There was a statistically significant difference in mean food security score at follow-up when controlling for baseline food security and looking only at post-COVID sites (*p* = 0.01; Cohen’s *d* = 0.4). VV users improved their food security score, through a reduction in 0.5 (0.2) points, whereas VV non-users’ food security scores worsened through an increase in 0.3 (0.2) points. Given that participation in government and food assistance programs was found to be different between COVID versus post-COVID in this sub-sample, we added these as covariates in food security models, but they did not alter the estimates or significance (*p* = 0.01).

**Table 5:** Impact of the Veggie Van on participants’ food security status in the Veggie Van study: comparison of Veggie Van users versus Veggie Van non-users

Food Security ^a^					
Outcome	Veggie Van Users ^b^ (*n* = 171) Mean (SE)	Veggie Van Non-Users (*n* = 369) Mean (SE)	Mean Difference (SE)	*p*-value	*n*
*Baseline* ^c^	2.3 (0.3)	2.0 (0.2)	0.2 (0.3)	0.46	539
*12-Month Follow-up* ^c^	1.6 (0.2)	1.8 (0.2)	-0.3 (0.3)	0.33	452
Change at 12-months ^c^					
	-0.6 (0.2)	-0.2 (0.2)	-0.4 (0.2)	0.08	451
Change at 12-months controlling for baseline covariates (food security, income, and race) ^c^					
	-0.5 (0.2)	-0.2 (0.1)	-0.4 (0.2)	0.14	388
Change at 12-months controlling for baseline covariates (food security, income, and race); post-COVID ^d^ sites only ^c^					
	-0.5 (0.2)	0.3 (0.2)	-0.8 (0.3)	0.01	215

^a^Food security score ranges from 0 to 10 with a lower score indicating high food security and a higher security indicating lower food security (food insecurity)

^b^Veggie Van users indicated at their 12-month follow-up survey that they have shopped at the mobile market site at least once since the market launch

^c^GLMM – generalized linear mixed model; GLMM model was adjusted for clustering within sites

^d^COVID = Coronavirus Disease

### Changes in food security status among food insecure participants

At baseline, 34.2% of intervention and 35.3% of control participants were considered food insecure (Table 1). Among the subgroup of individuals who were food insecure at baseline and also completed follow-up data collection (*n* = 147), 35.5% of control group participants became food secure at follow-up compared with 47.1% of intervention participants (Chi-Square = 1.97, *p* = 0.16). When we looked at only participants at post-COVID sites (*n* = 80), we saw a similar pattern where 29.1% of control vs. 49.0% of intervention participants were food secure at follow-up (Chi-Square = 3.12, *p* = 0.08). Among previously food insecure VV users (*n* = 52), 51.9% were food secure at follow-up compared with 35.8% of non-users (*n* = 93) (Chi-Square = 3.23, *p* = 0.07). Among previously food insecure VV users at post-COVID sites (*n* = 30), 60% become food secure at follow-up compared to 29.2% of previously food insecure non-users (*n* = 48) (Chi-Square = 7.25, *p* = 0.007).

### Psychosocial outcomes

Overall, there were not any statistically significant differences in any of the psychosocial measures (perceived access, self-efficacy or barriers) between baseline and follow-up based on intervention group or VV usage. Total self-efficacy at baseline was 55.7 (19.7) for the intervention group and 57.1 (18.7) for the control group (*p* = 0.29). This indicates that most participants felt that purchasing, eating and preparing more F&V would neither be very easy or very hard. At follow-up, there were no statically significant differences in scores between the groups (*p* = 0.75): intervention 57.3 (16.0) and control 59.0 (15.8). For perceived barriers, the total barriers score at baseline was 23.5 (5.1) for the intervention group and 22.5 (5.0) for the control group (*p* = 0.09). This equates to a neutral barrier score; a score of 22 would indicate on average not agreeing or disagreeing with stated barriers and higher scores indicate more agreement with barriers. At follow-up, there were no statically significant differences in total barrier scores between the groups (*p* = 0.63): intervention 23.1 (5.1) and control 22.6 (5.0). For perceived access around the home, mean scores at baseline were 9.3 (7.2) for the intervention group and 9.4 (6.5) for the control group (*p* = 0.81.). Nine is considered a neutral score (i.e., participants neither agree nor disagree that fresh F&Vs are available, high quality or varied around where they live). At follow-up, there were no statically significant differences in scores between the groups (*p* = 0.08): intervention 10.0 (6.5) and control 9.4 (5.9). Lastly, mean perceived affordability scores at baseline were 3.7 (1.7) for the intervention group and 3.4 (1.6) for the control group (*p* = 0.09). At follow-up, there were no statically significant differences in scores between the groups (*p* = 0.28): intervention 3.8 (1.6) and control 3.6 (1.5). A score of 3 is neutral (on a scale of 1 to 5) and a higher score means greater agreement that fresh F&V are affordable. Refer to supplementary materials 1–3 for psychosocial outcome results.

## Discussion

The goal of this research was to understand the impact of the VV model when delivered by multiple community organizations with minimal involvement from the research team. This was the first effectiveness study of a mobile market conducted across organizations and communities. It was also the first RCT of a mobile market to look at food insecurity as an outcome. This study’s intent-to-treat analyses did not observe any statistically significant differences between intervention and control groups for the main outcome of F&V consumption or the added outcome of food security. A main challenge with conducting RCTs in retail settings is that participants are required to make a purchase to get a dose of the intervention. Only 40.1% of participants at VV sites reported ever shopping at VV, compared to 63.5% in the efficacy study [26]. While the research team attempted to recruit participants who intended to shop at VV, many did not. In the efficacy study, purchasing was encouraged through weekly newsletters to participants from the VV as well as a free box of produce at their first visit. While partner organizations were encouraged to provide newsletters and incentives, most did not.

Given the challenges of ensuring intervention exposure, and the relationship between frequency of purchases and F&V consumption seen in previous research [25, 26], this study planned to look at the relationship between intervention exposure and the main outcomes. Unfortunately, most partner organizations did not reliably collect purchasing data, so a dose-response analysis was not possible. Instead, self-report data was used looking at whether participants had ever used VV (as a dichotomous variable). As in the efficacy study, we did not observe an association between ever shopping at VV and F&V consumption. We did, however, find that VV customers at post-COVID sites had higher food security at follow-up than participants that never used VV.

While previous mobile market researchers have suggested that mobile markets could impact food security by offering food at lower prices than traditional food retail [27], this is the first study to examine their impact on food security in a RCT. The findings suggest the need for further study of food security among VV users as VV was not intended as a food security intervention, but rather a multi-level dietary change intervention. This outcome was added based on formative work with mobile market operators who expressed that addressing food insecurity and food access issues were central to their mobile market’s mission [22]. A future study could consider looking at the impact of VV only among food insecure participants as only 35% of the current study sample were considered food insecure at baseline. While this rate is significantly higher than the national average [53], it did not represent the majority of participants. A challenge in measuring changes in food security among a group with mixed food security levels is that there is no standard analysis for evaluating change. The USDA food security measure was designed for population-level surveillance and is not well designed to capture change as responses are skewed toward 0 (high food security). To address this, we conducted an exploratory sub-group analysis among participants who were food insecure at baseline. These findings were in line with food security findings for the entire sample; notably, among the post-COVID sites, food security rates were more than double for VV users vs. non-users.

While the VV model was designed to offer fresh, local produce at affordable prices, it did not offer free food which is notable as prior to this, a key food security solution has been distribution of free food at food pantries. While distributing free food can be effective, food pantries face concerns with stigma, quality and sustainability [54] that may be overcome by a mobile market model [55]. It is also possible that VV enhances food security programs that supplement income (e.g., SNAP) by giving participants a convenient way to access incentive and matching programs (e.g., Double Up Food Bucks) for healthy foods that might otherwise only be available at farmers’ markets. As mobile market models evolve, many organizations are leveraging produce incentive or prescription programs that essentially render F&V free or significantly below market price for eligible customers but still offer the choice and quality customers are looking for [24].

Despite not observing statistically significant differences, changes in F&V intake at post-COVID sites are in line with previous VV studies where intervention participants had about a 0.5 cup/day increase in F&V consumption compared control sites (*p* = 0.43) [25, 26]. While not statistically significant in this sample, this change could be significant at a population level given low F&V intake among the target population [7]. The current study was affected by several limitations, most notably the COVID-19 pandemic that severely affected food retail [56]. The pandemic resulted in major fluctuations in food security and diet throughout 2020 and part of 2021, which may have inflated baseline food insecurity rates; further, hesitancy to shop in person combined with provision of additional SNAP benefits or free food during the intervention period may have made it harder to show change at follow-up. Although delayed-intervention control sites in this study had signed MOUs indicating that they would not start any new food or nutrition program, the extenuating circumstances of the pandemic necessitated flexibility. Thus, delayed-intervention control sites may have been exposed to similar food programs. There were also some intervention sites that decided to completely shut down mobile markets for some or all of 2020, or that significantly changed their model to comply with pandemic-related restrictions of their organization or municipality. While the analysis plan already included looking at differences in primary outcomes based on whether people reported shopping at VV or not, post-hoc analyses were conducted to only look at sites that started study participation after major COVID restrictions had been lifted.

In addition to the impact on mobile market operations, the pandemic also limited the ability to recruit participants due to both university and partner site shutdowns. This forced the study to pivot much of the recruitment online, rather than previously planned community events at the partner sites; this may have impacted both the type and number of participants that were recruited. However, from a demographic perspective, participants were similar to other low-income urban populations recruited in our previous research [25, 29]. In-person data collection (height, weight, and Veggie Meter readings) at several sites was cancelled leading to a greater reliance on self-reported measures such as BRFSS and food security for analyses. Lastly, multiple Veggie Meters were used among partner organizations and between baseline and follow-up timepoints. Not using the same device on participants between baseline and follow-up emerged as a potential threat to measurement validity after this study was conducted [57, 58]. Given that Veggie Meter scores are best interpreted as relative change and it is still unknown how they directly related to intake, we have concerns about the validity of this data. Further, dermal carotenoids only measure intake of carotenoid-rich F&Vs so many items offered at VV may not be reflected in these scores. Given the challenges with both 24-hour recall and Veggie Meter data, BRFSS outcomes were included for which there was more complete data collection even if this was not the planned, gold-standard outcome [37]. More research is needed to understand the impact of mobile markets on diet and food insecurity outside the context of a global pandemic. The research team is currently analyzing data from focus groups with VV users to better understand whether they are using VV to help increase F&V consumption through better access to reduce F&V expenditures, or both.

As with any community-engaged research working with multiple partners, the research team could not control all aspects of the study, including participant recruitment. Community sites and partner organizations worked together to collect interest forms which the research team used to recruit participants. The logistical reasons for using interest forms and recruiting participants after randomization are discussed in detail elsewhere [37]. One limitation which may have resulted from this approach was fewer interest forms were collected from delayed-intervention control sites. This may have been due to a lack of investment or urgency on the part of the sites/partners as they did not have an imminent deadline (i.e., a market start) by which they had to collect interest forms. Despite lower numbers in the control group, recruitment rates were similar across groups with retention being slightly higher in the control group; retention rates are also in line with other community based studies [59]. Thus, while this approach may have affected the pool of available participants, it did not bias enrollment. An additional limitation of this approach was that not all intervention participants were able to complete 24-hour recalls (if baseline data collection continued past market launch), limiting the power for this outcome.

Another potential limitation to VV effectiveness was incomplete implementation of the intervention. Preliminary implementation analyses indicate that none of the original partners reported high fidelity to the VV model. This may have been because partners did not fully understand how to implement certain components, such as bundles, or they were unable to implement certain aspects, like nutrition/food education, due to COVID-19 or other local restrictions. To augment the sample size following pandemic-related challenges, new partners were recruited in 2021 and 2022. These additional partners received more intensive training on the VV model in an effort to improve implementation [60]. This additional training could partially explain why some outcomes improved among post-COVID sites. Future publications will examine implementation of the model at each site and its relationship with changes in primary outcomes; this will also help elucidate which parts of the VV model are necessary to have a positive impact.

While we hypothesized that the VV increases F&V intake by addressing the 5 A’s of food access (availability, accessibility, affordability, acceptability, and accommodation) [37, 38], the results indicate that participants’ perceptions of food access or affordability in their community were unchanged. Previous research suggests that availability, affordability, and accessibility are necessary but not sufficient for change and that the most effective studies address all 5 A’s of food access and include food education [16, 26]. In the previous efficacy study, there were improvements in self-efficacy for buying and preparing F&V for the intervention group; however, the current study did not show changes in self-efficacy, likely due to poor implementation of the nutrition/food education components of the intervention. This may also help explain why there was not a significant change in F&V intake in the intent-to-treat analysis. Qualitative feedback from ongoing VV research indicates that the presence of VV in a community and the VV educational components that extend beyond the market may prompt better dietary behaviors even among non-users.

## Conclusions

This is the first study to show that people who use mobile markets may improve their food security scores. Contrary to previous research, we did not observe statistically significant diet-related changes, however, the COVID pandemic introduced numerous challenges in terms of implementation and participant engagement. Together with previous research showing the acceptability of mobile markets, these findings suggest the potential benefits of mobile markets to address disparities in access to healthy food within lower-income and underserved communities. As mobile markets expand nationwide, more research is needed to understand which practices improve reach, sustainability, and impact. Future research should consider using high quality natural experiments to evaluate mobile market impact, given the challenges with ensuring dosage in a RCT of a food retail intervention [61].

## Supplementary Information

Supplementary Material 1. Supplementary Table 6.

Supplementary Material 2. Supplementary Table 7.

Supplementary Material 3. Supplementary Table 8.

Supplementary Material 4. Veggie Van Baseline Survey.

Supplementary Material 5. Veggie Van Follow-Up Survey.

## Data Availability

The datasets used and/or analyzed during the current study are available from the corresponding author on reasonable request.

## Abbreviations

BRFSS: Behavioral Risk Factor Surveillance Survey
COVID-19: Coronavirus disease 2019
F&V: Fruit and Vegetable
GLMM: Generalized linear mixed model
NDSR: Nutrition Data Systems for Research
RCT: Randomized Controlled Trial
SNAP: Supplemental Nutrition Assistance Program
TANF: Temporary Assistance for Needy Families
US: United States
USDA: United States Department of Agriculture
VV: Veggie Van
WIC: Special Supplemental Nutrition Program for Women, Infants, and Children
