# Supplementary material for: Cluster-randomized controlled trial of a mobile produce market designed to address diet and food insecurity in underserved communities
Source: BMC Nutr. 2026 Apr 9;12:94. doi: 10.1186/s40795-026-01302-7 (PMC13182037; doi:10.1186/s40795-026-01302-7)
Supplement: Supplementary file 1 — Supplementary Material 1. Supplementary Table 6. [file 40795_2026_1302_MOESM1_ESM.docx]

**Supplementary Table 6: Impact of the Veggie Van on Participants’ Perceptions of Food Access in the Veggie Van Study**

| **Outcome** | **Intervention  (n=426)** | **Control  (n=273)** | **Intervention Effect** | **P value** | **n** |
| --- | --- | --- | --- | --- | --- |
|  | **Mean (SE)** | **Mean (SE)** | **Mean Difference (SE)** |  |  |
| **General Perceived Access ^a^** | | | | | |
| Change at 12-months ^b^ | 0.4 (0.2) | 0.3 (0.2) | 0.2 (0.3) | 0.59 | 456 |
| **Perceived Access around Veggie Van Site ^c^** | | | | | |
| Change at 12-months ^b^ | 0.8 (0.3) | 0.01 (0.4) | 0.8 (0.5) | 0.12 | 437 |
| **Perceived Access around Home ^d^** | | | | | |
| Change at 12-months ^b^ | 0.8 (0.2) | 0.1 (0.3) | 0.6 (0.3) | 0.08 | 448 |
| **Affordability of Fruits and Vegetables ^e^** | | | | | |
| Change at 12-months ^b^ | 0.1 (0.1) | 0.2 (0.1) | -0.1 (0.1) | 0.28 | 465 |

^a^ The general perceived access scale assesses participants’ perceptions of access to fruits and vegetables in general. A higher score indicates favorable perceptions of access. A lower score indicates less favorable perceptions of access.

^b^ GLMM = generalized linear mixed model; GLMM model was adjusted for clustering within sites

^c^ The perceived access around Veggie Van Site scale assesses participants’ perceptions of access to fruits and vegetables around the community site that hosts the mobile market. A higher score indicates favorable perceptions of access. A lower score indicates less favorable perceptions of access.

^d^ The perceived access around home scale assesses participants’ perceptions of access to fruits and vegetables in their neighborhood. A higher score indicates favorable perceptions of access. A lower score indicates less favorable perceptions of access.

^e^ The affordability of fruits and vegetables scale assess participants’ perceptions of affordability of fruits and vegetables. A higher score indicates favorable perceptions of affordability. A lower score indicates less favorable perceptions of affordability.
