# Supplementary material for: Cluster-randomized controlled trial of a mobile produce market designed to address diet and food insecurity in underserved communities
Source: BMC Nutr. 2026 Apr 9;12:94. doi: 10.1186/s40795-026-01302-7 (PMC13182037; doi:10.1186/s40795-026-01302-7)
Supplement: Supplementary file 2 — Supplementary Material 2. Supplementary Table 7. [file 40795_2026_1302_MOESM2_ESM.docx]

**Supplementary Table 7: Impact of the Veggie Van on Participants’ Self-efficacy in the Veggie Van Study**

| **Self-Efficacy ^a^ Item** | **Intervention  (n=426)** | **Control  (n=273)** | **Intervention Effect** | **P value** | **n** |
| --- | --- | --- | --- | --- | --- |
|  | **Mean Change at 12-months (SE) ^b^** | **Mean Change at 12-months (SE) ^b^** | **Mean Difference (SE) ^b^** |  |  |
| 1. How easy or hard would it be for you to buy more fruits and vegetables than you normally do the next time you shop? | 0.3 (0.2) | 0.2 (0.3) | 0.1 (0.3) | 0.74 | 457 |
| 2. How easy or hard would it be for you to use all of the fruits and vegetables that you buy before they go bad? | -0.004 (0.2) | -0.02 (0.2) | 0.02 (0.3) | 0.97 | 463 |
| 3. How easy or hard would it be for you to work more fruits and vegetables than you normally do into meals for yourself and your family? | 0.05 (0.2) | -0.05 (0.2) | 0.1 (0.3) | 0.73 | 456 |
| 4. How easy or hard would it be for you to work more fruits and vegetables than you normally into snacks for yourself and your family? | 0.02 (0.2) | -0.01 (0.2) | 0.03 (0.3) | 0.93 | 457 |
| 5. How easy or hard would it be for you to cook vegetables in a way that is appealing to your family? | 0.2 (0.2) | 0.3 (0.2) | -0.1 (0.3) | 0.77 | 442 |
| 6. How easy or hard would it be for you to make-up a vegetables dish with what you have on hand? | 0.3 (0.2) | 0.3 (0.3) | 0.04 (0.9) | 0.97 | 461 |
| 7. How easy or hard would it be for you to try vegetables that you have not eaten before?” | -0.004 (0.2) | -0.01 (0.3) | 0.01 (0.4) | 0.98 | 456 |
| 8. How easy or hard would it be for you to prepare and cook new recipes? | 0.4 (0.2) | 0.07 (0.3) | 0.4 (0.3) | 0.29 | 458 |
| **Self-efficacy Total** | 0.8 (1.0) | 1.4 (1.3) | -0.5 (1.7) | 0.75 | 417 |

^a^ The self-efficacy scale assesses participants’ confidence in preparing and consuming fruits and vegetables. A higher score indicates higher self-efficacy. A lower score indicates lower self-efficacy.

^b^ GLMM – generalized linear mixed model; GLMM model was adjusted for clustering within sites
