# Supplementary material for: Cluster-randomized controlled trial of a mobile produce market designed to address diet and food insecurity in underserved communities
Source: BMC Nutr. 2026 Apr 9;12:94. doi: 10.1186/s40795-026-01302-7 (PMC13182037; doi:10.1186/s40795-026-01302-7)
Supplement: Supplementary file 3 — Supplementary Material 3. Supplementary Table 8. [file 40795_2026_1302_MOESM3_ESM.docx]

**Supplementary Table 8: Impact of the Veggie Van on Participants’ Perceptions of Barriers to Consuming Fruits and Vegetables in the Veggie Van Study**

| **Perceived Barrier ^a^ Item** | **Intervention  (n=426)** | **Control  (n=273)** | **Intervention Effect** | **P value** | **n** |
| --- | --- | --- | --- | --- | --- |
|  | **Mean (SE)** | **Mean (SE)** | **Mean Difference (SE)** |  |  |
| **Ease of eating F&V** | | | | | |
| Change at 12-months ^b^ | -0.02 (0.1) | 0.02 (0.1) | - 0.04 (0.1) | 0.64 | 459 |
| **Time to prepare F&V** | | | | | |
| Change at 12-months ^a^ | -0.1 (0.1) | 0.1 (0.1) | -0.1 (0.1) | 0.17 | 454 |
| **Knowledge to prepare F&V** | | | | | |
| Change at 12-months ^b^ | -0.1 (0.04) | -0.1 (0.1) | -0.01 (0.1) | 0.85 | 461 |
| **Transportation to get F&V** | | | | | |
| Change at 12-months ^b^ | -0.01 (0.04) | -0.03 (0.1) | 0.02 (0.1) | 0.76 | 459 |
| **Cost of F&V** | | | | | |
| Change at 12-months ^b^ | -0.1 (0.1) | -0.1 (0.1) | 0.002 (0.1) | 0.98 | 438 |
| **Taste preference for fruit** | | | | | |
| Change at 12-months ^b^ | 0.03 (0.04) | 0.03 (0.04) | 0.005 (0.1) | 0.93 | 463 |
| **Taste preference for vegetables** | | | | | |
| Change at 12-months ^b^ | -0.04 (0.04) | -0.01 (0.05) | -0.03 (0.1) | 0.59 | 465 |
| **Family taste preference for fruit** | | | | | |
| Change at 12-months ^b^ | 0.03 (0.1) | 0.01 (0.1) | 0.02 (0.1) | 0.84 | 359 |
| **Family taste preference for vegetables** | | | | | |
| Change at 12-months ^b^ | -0.004 (0.1) | 0.02 (0.1) | -0.02 (0.1) | 0.82 | 356 |
| **Space to store F&V** | | | | | |
| Change at 12-months ^b^ | -0.02 (0.1) | 0.1 (0.1) | -0.1 (0.1) | 0.34 | 458 |
| **Restaurants offering F&V** | | | | | |
| Change at 12-months ^b^ | -0.07 (0.1) | 0.008 (0.1) | -0.1 (0.1) | 0.41 | 388 |
| **Workplace having F&V** | | | | | |
| Change at 12-months ^b^ | -0.2 (0.1) | -0.1 (0.1) | -0.02 (0.2) | 0.91 | 207 |
| **Total Barriers Score** | | | | | |
| Change at 12-months ^b^ | -0.3 (0.7) | 0.2 (0.9) | -0.6 (1.1) | 0.63 | 145 |

^a^ The perceived barriers scale assesses participants’ perception of barriers to accessing, preparing, and consuming fruits and vegetables. A higher score indicates stronger perceptions of barriers. A lower score indicates weaker perceptions of barriers.

^b^ GLMM – generalized linear mixed model; GLMM model was adjusted for clustering within sites
