# Supplementary material for: Cluster-randomized controlled trial of a mobile produce market designed to address diet and food insecurity in underserved communities
Source: BMC Nutr. 2026 Apr 9;12:94. doi: 10.1186/s40795-026-01302-7 (PMC13182037; doi:10.1186/s40795-026-01302-7)
Supplement: Supplementary file 4 — Supplementary Material 4. Veggie Van Baseline Survey. [file 40795_2026_1302_MOESM4_ESM.pdf]

Veggie Van Baseline Survey

SECTION 1: Perceived Access

Please think about the places that you can buy groceries in the neighborhood near where you live. Think of your neighborhood as the area within a 20-minute walk or about one mile from your home. For each of the following statements, please tell me whether you strongly agree, agree, feel neutral, disagree, or strongly disagree.
[NOTE TO INTERVIEWER:] for questions in the following block, only read the response options if the participant cannot remember the answer choices

	Strongly Agree	Agree	Feel Neutral	Disagree	Strongly Disagree	Do Not Know	Skip

1.1 It is easy to buy fresh fruits and vegetables in my neighborhood.	◯	◯	◯	◯	◯	◯	◯
1.2 There is a large selection of fruits and vegetables in my neighborhood.	◯	◯	◯	◯	◯	◯	◯
1.3 The fresh fruits and vegetables in my neighborhood are high quality	◯	◯	◯	◯	◯	◯	◯

PERCEIVED ACCESS
Please think about the places that you can buy groceries around [site]. Think of the area within a 20-minute walk or about one mile from [site]. For each of the following statements, please tell me whether you strongly agree, agree, feel neutral, disagree, or strongly disagree.
[NOTE TO INTERVIEWER:] for questions in the following block, only read the response options if the participant cannot remember the answer choices.

	Strongly Agree	Agree	Feel Neutral	Disagree	Strongly Disagree	Do Not Know	Skip
1.4 It is easy to buy fresh fruits and vegetables around [site].	◯	◯	◯	◯	◯	◯	◯
1.5 There is a large selection of fruits and vegetables available around [site].	◯	◯	◯	◯	◯	◯	◯
1.6 The fresh fruits and vegetables around [site] are high quality.	◯	◯	◯	◯	◯	◯	◯

PERCEIVED ACCESS
Now, please think about your ability to find and purchase fresh fruits and vegetables in general. Again please tell me whether you strongly agree, agree, feel neutral, disagree, or strongly disagree.
[NOTE TO INTERVIEWER:] for questions in the following block, only read the response options if the participant cannot remember the answer choices

	Strongly Agree	Agree	Feel Neutral	Disagree	Strongly Disagree	Do Not Know	Skip
1.7 It is easy for me to get to a place where I can buy fresh fruits and vegetables.	◯	◯	◯	◯	◯	◯	◯
1.8 I can afford to buy enough fresh fruits and vegetables for myself or my family.	◯	◯	◯	◯	◯	◯	◯

1.9 It is easy for me to find a variety of fresh fruits and vegetables.	◯	◯	◯	◯	◯	◯	◯
1.10 It is easy for me to find high quality fresh fruits and vegetables.	◯	◯	◯	◯	◯	◯	◯

Compared to before the COVID-19 or Coronavirus pandemic, is it easier or harder to find fresh fruits and vegetables?

- ◯ It is about the same as before
- ◯ It has gotten easier to find fresh fruits and vegetables
- ◯ It has gotten harder to find fresh fruits and vegetables

SECTION 2: Participant Demographics & Body Composition

The next group of questions will ask about you and your family. Just a reminder, any individual information you provide during this survey will be kept completely private. You are not required to answer any questions that you don't want to. If you would like to skip any question, just say "I choose not to answer this question, "and I will move on.

DEMOGRAPHICS
2.1 What is your gender?
[Note to interviewer: Do Not Read choices.

- ◯ Male
- ◯ Female
- ◯ Other
- ◯ Skip

DEMOGRAPHICS
2.2 What is your birthdate? (month-day-year) ________________________________

BODYCOMP
2.3 How tall are you?
(inches) Note to interviewer: Do not read answer choices. If participant is having trouble answering, ask for his/her best guess.

If participant gives you feet + inches, i.e. "I'm 5 foot 10" use following table:

4'6" = 54 inches
4'7" = 55 inches
4'8" = 56 inches
4'9" = 57 inches
4'10" = 58 inches
4'11" = 59 inches
5'0" = 60 inches
5'1" = 61 inches
5'2" = 62 inches
5'3' = 63 inches
5'4" = 64 inches
5'5" = 65 inches
5'6" = 66 inches
5'7" = 67 inches
5'8" = 68 inches
5'9" = 69 inches
5'10" = 70 inches
5'11" = 71 inches
6'0" = 72 inches
6'1" = 73 inches
6'2" = 74 inches
6'3" = 75 inches
6'4" = 76 inches
6'5" = 77 inches
6'6" = 78 inches
6'7" = 79 inches
6'8" = 80 inches
6'9" = 81 inches
6'10" = 82 inches
6'11" = 83 inches
7' 0" = 84 inches

____

BODYCOMP
2.4 How much do you weigh right now? (in POUNDS)
[Note to interviewer: If participant is having trouble answering, ask for his/her best guess.]

____

BODYCOMP:
Height in centemeters (cm)

____

BODYCOMP: Weight in kilograms (kg)

____

BODYCOMP:
Body Mass Index (BMI)

____

DEMOGRAPHICS<br>2.5 What is your current marital status?<br><br>[Note to interviewer:] Don't read answer choices.	◯ Married or living with a partner<br>◯ Single<br>◯ Divorced<br>◯ Separated<br>◯ Widowed<br>◯ Skip
DEMOGRAPHICS<br>2.6 What is the highest level of education you have completed?<br><br>[Note to Interviewer:] Don't read choices unless participant is having trouble.	◯ 8th Grade or less<br>◯ Some high school<br>◯ High School graduate or GED<br>◯ Trade or beauty school graduate<br>◯ Some College<br>◯ College graduate<br>◯ More than college (Some post graduate, post graduate, or professional degree<br>◯ Skip
DEMOGRAPHICS<br>2.7 Which of the following best describes your yearly household income?<br>[Note to Interviewer:] This question is asking for the yearly household income, not just the income of the participant.	◯ Lesss than $10,000<br>◯ $10,000 to $19,999<br>◯ $20,000 to $29,999<br>◯ $30,000 to $39,999<br>◯ $40,000 to $49,999<br>◯ $50,000 to $59,999<br>◯ $60,000 or more<br>◯ Skip
What is your current employment status?	◯ Working full-time<br>◯ Working part-time<br>◯ Retired<br>◯ SSI/Disability<br>◯ Not currently working<br>◯ Working a contract or temporary position<br>◯ Working a gig job (delivery, uber, etc.)<br>◯ Skip
Has your employment status changed since the COVID-19 or Coronavirus pandemic started? [select all that apply]	☐ Yes, I was laid off<br>☐ Yes, I quit my job<br>☐ Yes, I am working fewer hours<br>☐ Yes, I have been furloughed<br>☐ Yes, I have been required to work from home<br>☐ Yes, I am working more hours<br>☐ Yes, I changed jobs<br>☐ No, I have not had any job changes<br>☐ I don't work, not applicable<br>☐ Skip
DEMOGRAPHICS<br>2.8 Do you consider yourself Hispanic or Latino/Latina?	◯ Yes- HISPANIC/LATINO/LATINA<br>◯ No<br>◯ Skip
DEMOGRAPHICS<br>2.9A What race(s) do you consider yourself? May select as many as applicable.<br><br>Note to Interviewer: Do not read answer choices.	☐ American Indian or Alaskan Native<br>☐ Asian<br>☐ Black/African American<br>☐ Native Hawaiian or Other Pacific Islander<br>☐ White<br>☐ Other (Specify)<br>☐ Skip
DEMOGRAPHICS<br>2.9A Specify what "Other Race" you consider yourself.	______________________________

DEMOGRAPHICS<br>2.9B Among the following choices, what race do you most identify with? (single answer)	◯ American Indian or Alaskan Native<br>◯ Asian<br>◯ Black/African American<br>◯ Hispanic or Latino/Latina<br>◯ Native Hawaiian or Other Pacific Islander<br>◯ White<br>◯ Other (Specify)<br>◯ Skip
DEMOGRAPHICS<br>2.9B Specify what "Other Race" do you most identify with.	__________________________________
DEMOGRAPHICS<br>2.10 I'm going to list the names of some programs. Please tell me if you or anyone in your household has participated in any of the following programs within the past 12 months. [Note to Interviewer: check ALL that apply.]<br><br>Note to interviewer: Temporary Assistance for Needy Families or Welfare may be referred to as TANF or cash	☐ WIC<br>☐ SNAP (Food Stamps)<br>☐ Free or reduced-price school breakfast or lunch<br>☐ Head Start<br>☐ Food Pantry<br>☐ Medicaid<br>☐ Temporary Assistance for Needy Families or Welfare (or TANF or cash)<br>☐ Social Security Disability Benefits<br>☐ None<br>☐ Skip
Were you or anyone in your household eligible for Pandemic EBT or P-EBT benefits in the past year?	◯ Yes and we used it to purchase food for our household<br>◯ Yes, but we did not use it to purchase food for our household<br>◯ No<br>◯ Don't know<br>◯ Skip
In the past year, did you or anyone in your household receive boxes of food through the USDA Farm to Families Food Box program, which included distribution of free farm products like Fruits & Vegetables, or meat, or dairy?<br>Note to interviewer: not food pantry	◯ Yes, We received 1-2 boxes<br>◯ Yes, We received 3-4 boxes<br>◯ Yes, we received 5 or more boxes<br>◯ No, we did not receive any boxes
Did you or anyone in your household receive unemployment in the past year?	◯ Yes<br>◯ No<br>◯ Don't know<br>◯ Skip
DEMOGRAPHICS<br>2.11 How long have you resided at your current location? (IF more than 1 year, enter in whole years).<br><br>Note to Interviewer: ENTER data in YEARS.<br>For residency < 1 year:<br>1 month = .08 | 2 months = .16<br>3 months = .25 | 4 months = .33<br>5 months = .41 | 6 months = .5<br>7 months = .58 | 8 months = .66<br>9 months = .74 |10 months = .82<br>11 months = .90	__________________________________

DEMOGRAPHICS
2.12 How many adults for whom you buy food live in your household? Please include all adults 18 years of age or older, including yourself, in this number.

____

DEMOGRAPHICS
2.13 How many children for whom you buy food live in your household? Please include all children younger than 18 years of age in this number.

____

DEMOGRAPHICS
Mouths to feed total

____

Section 3 HDQ: Health and Diet Questions (partial)

The next questions are about your health.

3.1. In the past 12 months, has a health care provider told you that you have either of the following conditions?

	yes	no	don't know	skip
High blood pressure	○	○	○	○
Diabetes	○	○	○	○

HDQ
3.2 How do you manage your Diabetes (choose all that apply)

- ☐ diet
- ☐ oral medication
- ☐ insulin
- ☐ other

HDQ
3.2a. Please specify the "OTHER" methods you use to manage your diabetes.

____

HDQ
3.3 Are you currently following a special diet?

- ○ yes
- ○ no
- ○ don't know/skip

HDQ
3.3a. What type of special diet?

- ☐ for diabetes
- ☐ low fat
- ☐ low salt
- ☐ weight loss
- ☐ vegetarian
- ☐ gluten free
- ☐ other (specify)
- ☐ don't know

HDQ
3.3b. What type of OTHER diet?

____

HDQ
3.4. In general, would you say your health is:

- ○ Excellent
- ○ Very good
- ○ Good
- ○ Fair
- ○ Poor
- ○ Skip/Refused

SECTION 4: DIET MEASURES: FRUITS AND VEGETABLES

FOR PHASE 1: Now, I want you to think about what you usually ate in the last month. Please think about ALL
the fruits and vegetables that you ate last month.

FOR PHASE 2: Now, I want you to think about what you usually ate in the month before a mobile market was brought to [site]. Please think about ALL
the fruits and vegetables that you ate last month.

Include those that were:
raw and cooked.
eaten as snacks and as meals.
eaten at home and away from home (at restaurants, friends' houses, and/or take-out
and eaten alone and mixed with other foods

4.1 Thinking about ALL the vegetables you ate over the last month -- On average, how many servings of vegetables did you usually eat each day? Consider one serving of vegetables to be one medium-sized whole vegetable (about the size of a baseball), one cup of chopped or canned vegetables, or two cups of raw, leafy greens or lettuce.

[Note to Interviewer:] Enter the number of servings (in whole numbers) that the participant reports eating on the line provided. If the participant responds with "Less than one serving", enter 0.

◯ Number of Servings
◯ Do Not Know
◯ Skip

3.2 Number of Vegetable Servings

____

3.3 Thinking about ALL the fruits you ate over the past month -- on average, how many servings of fruit did you usually eat each day?

Consider one serving of fruit to be one medium-sized whole fruit (about the size of a baseball), or one cup of chopped or canned fruit.

Include only fresh, frozen, adn/or canned fruits, but not fruit juice.

◯ Number of Servings
◯ Do Not Know
◯ Skip

4.4 Number of Fruit Servings

____

Compared to before the COVID-19 or Coronavirus pandemic, have you changed how often you eat fresh fruits and vegetables?

◯ No, it's about the same as before the pandemic
◯ Yes, I am eating more fresh fruits and vegetables
◯ No, I am eating fewer fresh fruits and vegetables

SECTION 5: BARRIERS

The next questions are about reasons that some people may find it easy or hard to eat enough fruits and vegetables. For each of the following statements, please tell me whether you strongly agree, agree, disagree, or strongly disagree.

Note to Interviewer:

For the following statements, only read each response options if the participant cannot remember the answer choices. If a statement does not apply to a participant (e.g. The participant does not work, and therefore cannot answer the statement about workplace availability , OR The participant lives alone and does not provide food for a family and therefore cannot answer the statement about the family's liking of fruits or vegetables), then choose "SKIP" for that statement.

	Strongly Agree	Agree	Disagree	Strongly Disagree	Do Not Know	Skip
1. It is easy for me to eat fruits and vegetables.	○	○	○	○	○	○
2. I do not have time to prepare fruits and vegetables.	○	○	○	○	○	○
3. I do not know how to prepare fruits and vegetables.	○	○	○	○	○	○
4. I do not have transportation to get to a place where I can get fruits and vegetables.	○	○	○	○	○	○
5. It cost too much money to buy fruits and vegetables.	○	○	○	○	○	○
6. I do not like fruits.	○	○	○	○	○	○
7. I do not like vegetables.	○	○	○	○	○	○
8. My family does not like fruits.	○	○	○	○	○	○
9. My familiy does not like vegetables	○	○	○	○	○	○
10. I do not have enough space to store fruits and vegetables in my home.	○	○	○	○	○	○
11. The restaurants I go to do not offer fruits and vegetables.	○	○	○	○	○	○
12. Fruits and vegetables are not available at my work (in the cafeteria or other vending outlets).	○	○	○	○	○	○

13. I cannot afford to buy organic vegetables	○	○	○	○	○	○
14. I cannot afford to buy organic fruit	○	○	○	○	○	○

Next, I am going to ask you about some ways that the Covid-19 pandemic may have made it easier or harder to buy fresh fruits and vegetables. Again you can answer strongly agree, agree, disagree, or strongly disagree.

	Strongly Agree	Agree	Disagree	Strongly Disagree	Do Not Know	Skip
Fruits and vegetables have gotten more expensive since the pandemic started	○	○	○	○	○	○
I am not shopping as often and it is difficult to keep fresh fruits and vegetables on hand until the next time I shop	○	○	○	○	○	○
I'm concerned about the safety of public transportation, so I'm not shopping as much	○	○	○	○	○	○
I'm concerned that fresh fruits and vegetables are not safe to eat	○	○	○	○	○	○

BARRIERS:
reverse scoring for barriers item 1 ________________________________

BARRIERS 1 scoring ________________________________

BARRIERS 2 scoring ________________________________

BARRIERS 3 scoring ________________________________

BARRIERS 4 scoring ________________________________

BARRIERS 5 scoring ________________________________

BARRIERS 6 scoring ________________________________

BARRIERS 7 scoring ________________________________

BARRIERS 8 scoring ________________________________

BARRIERS 9 scoring ________________________________

BARRIERS 10 scoring

____

BARRIERS 11 scoring

____

BARRIERS 12 scoring

____

BARRIERS TOTAL SCORE

____

SECTION 6: SHOPPING

This next set of questions will ask you about shopping for food.

6.1 How Often do you usually shop for food?

Note to Interviewer: Don't read answer choices unless participant is having trouble answering.

- ◯ More than once a week
- ◯ Once a week
- ◯ Once every 1-2 weeks
- ◯ Once a month
- ◯ Less than once a month
- ◯ Do Not Know
- ◯ Skip

Compared to before the Coronavirus or COVID-19 pandemic, have you changed how often you usually shop for food?

- ◯ No, it's about the same as before the pandemic
- ◯ Yes, I am shopping more often
- ◯ Yes, I am shopping less often

In the past year, have you purchased groceries online?

If so, about how often?

- ◯ No
- ◯ Yes, a few times in the past year
- ◯ Yes, around once per month
- ◯ Yes, around once per week or almost once per week

Since the start of the coronavirus pandemic, have you changed how often you buy groceries online?

- ◯ No, it's about the same as before
- ◯ I am buying groceries online more often
- ◯ I am buying groceries online less often
- ◯ Other

Explain

____

6.2 At which type of store do you buy most of your food?<br>-----------------<br>[FOR INTERVIEWER:<br>If it is a market site that has already launched, SAY: "Think of a typical month before you started shopping at the mobile market"<br><br>Supermarket: Sells fresh meat (e.g., beef, pork, chicken, turkey) AND Has 4 or more cash registers AND Has at least two of the following service counters: Bakery, Butcher, or Deli<br><br>Grocery store: Sells fresh meat (e.g., beef, pork, chicken, turkey) AND Does not meet all the criteria for supermarket]. Examples of grocery stores include Trader Joe's, Aldi, Save A Lot and some ethnic and "mom-and-pop" food stores<br><br>Convenience Store - This includes small, independently-owned or chain stores that sell an edited selection of staple groceries and other convenience items, i.e., ready-to-heat and ready-to-eat foods. They often sell fresh milk and may have a deli or sell some processed meats (hot dogs, cold cuts, etc.) and other hot foods. Convenience stores are typically open long hours and some sell gasoline as well.]	◯ Supermarket or large grocery store ([region:value])<br>◯ Super-Center (Like Walmart or Target)<br>◯ Buying Club (like Sam's Club)<br>◯ Small grocery store (like Aldi)<br>◯ Dollar store<br>◯ Corner store, gas station, or convenience store<br>◯ Farmers Market, farm stand, or CSA<br>◯ Mobile produce market<br>◯ Other (Specify below)<br>◯ Do Not Know<br>◯ Skip
At which type of store do you buy most of your food? Specify Other	__________________________________
6.3 What are the names of the two places where you buy most of your food?<br><br>PLACE 1:	__________________________________
6.3 Place 2	__________________________________
6.4 Between [buystore1] and [buystore2] which store do you think you buy the most food from?	◯ Place 1 [buystore1]<br>◯ Place 2 [buystore2]
6.5 How do you usually shop for food from [buystore1]? Do you usually...	◯ Shop in person<br>◯ Shop online and have it delivered to your home<br>◯ Shop online and pick it up outside the store<br>◯ Other
Please specify other.	____
How do you usually shop for food from [buystore2]? Do you usually...	◯ Shop in person<br>◯ Shop online and have it delivered to your home<br>◯ Shop online and pick it up outside the store<br>◯ Other
Please specify other.	____

6.5 How do you usually travel to [buymainstore]?	◯ Walk<br>◯ Bicycle<br>◯ Bus or other public transportation<br>◯ Drive a car or other motor vehicle.<br>◯ Get a ride with someone else<br>◯ Taxi<br>◯ Other (Please Specify)<br>◯ Do Not Know<br>◯ Skip
6.5 How do you usually travel to [buymainstore]?<br>Specify Other mode of transport.	__________________________________
6.6 How long would it take you to get from your home to [buymainstore] if you walked there?<br><br>Note to interviewer: Don't read answer choices unless participant is having trouble.	◯ 10 minutes or less<br>◯ 11-20 minutes<br>◯ 21-30 minutes<br>◯ Do not know walk time, but I do know drive time (Specify)<br>◯ More than 30 minutes<br>◯ Do Not Know<br>◯ Skip
6.6 How long does it take to drive to [buymainstore]?	__________________________________
6.7 Where is [buymainstore]?<br><br>Please be as specific as you are able, giving the town, street, and/or nearby landmarks if possible.	__________________________________
6.8 At [buymainstore], how would you rate the price of fresh fruits and vegetables?	◯ Very affordable<br>◯ Somewhat affordable<br>◯ Somewhat expensive<br>◯ Very expensive<br>◯ Do Not Know<br>◯ Skip
6.9 At [buymainstore] how would you rate the quality of fresh fruits and vegetables?	◯ Very high quality<br>◯ Somewhat high quality<br>◯ Somewhat low quality<br>◯ Very low quality<br>◯ Do Not Know<br>◯ Skip
6.10 At [buymainstore] how would you rate the variety of fresh fruits and vegetables?	◯ Very high variety<br>◯ Somewhat high variety<br>◯ Somewhat low variety<br>◯ Very low variety<br>◯ Do Not Know<br>◯ Skip

6.11. On a scale of very important, somewhat important, a little important, not at all important:
How important are each of the following factors in your decision to shop at ([buymainstore])?

	Not at all important	A little important	Somewhat important	Very important	Do Not Know	Skip
a. Near your home	○	○	○	○	○	○
b. Near or on the way to other places where you spend time	○	○	○	○	○	○
c. Your friend/relatives shop at this store	○	○	○	○	○	○
d. Selection of foods	○	○	○	○	○	○
e. Quality of foods	○	○	○	○	○	○
f. Prices of foods	○	○	○	○	○	○
g. Access to public transportation	○	○	○	○	○	○
h. They offer an online shopping option	○	○	○	○	○	○

6.12a I'm going to read you a list of different types of stores. Please tell me how often you shop for fresh fruits and vegetables at each of the following types. When you answer, all you have to say is more than once a week, once a week, once every 1-2 weeks, once a month, rarely, or never.

Note to Interviewer: If the site is a market site that has already launched, SAY: "Think of a typical month before you started shopping at the mobile market".
For this section, only read answer choices if participant is having trouble answering.

(MAIN QUESTION)
How often do you buy fresh fruit and vegetables at...

	More than once a week	Once a week	Once every 1-2 weeks	Once a month	Rarely	Never	Do Not Know	Skip
...a supermarket (like [region:value])?	○	○	○	○	○	○	○	○
...a supercenter (like Walmart or Target)?	○	○	○	○	○	○	○	○
...a buying club (like Sam's Club, BJ's Warehouse, or Costco)	○	○	○	○	○	○	○	○
...a dollar store?	○	○	○	○	○	○	○	○
... a small grocery store (like Aldi)?	○	○	○	○	○	○	○	○

...a corner store, gas station, or convenience store?	◯	◯	◯	◯	◯	◯	◯	◯
...a farmers market, farm stand, or CSA	◯	◯	◯	◯	◯	◯	◯	◯
...mobile produce market?	◯	◯	◯	◯	◯	◯	◯	◯
...and other type of store?	◯	◯	◯	◯	◯	◯	◯	◯

6.12b Please specify the other type of store that you shop at for fresh fruits and vegetables. ________________________________

6.13 What are the names of the two places where you buy most of your fresh fruits and vegetables? ________________________________

(Place 1)

6.13b {What are the names of the two places where you buy most of your fresh fruits and vegetables?} ________________________________

(Place 2)

SECTION 7: COOKING

COOKING
7.1 In a typical week, how many days do you eat a main meal that is prepared at home?

Notes to Interviewer: Do not read answer choices. If the participant's answer falls between two of the choices listed below, read the two answer choices to the participant and ask them to pick which is the most correct.

If needed say "A main meal is the most substantial or most filling meal of your day".

- ◯ Never -- I do not prepare any main meals at home
- ◯ 1-2 days per week
- ◯ 3-4 days per week
- ◯ 5-6 days per week
- ◯ Every day
- ◯ Do Not Know
- ◯ Skip

Compared to before the Coronavirus or COVID-19 pandemic, have you changed how often you usually cook at home?

- ◯ No, it is about the same as before the pandemic
- ◯ Yes, I am cooking at home more often
- ◯ Yes, i am cooking at home less often

Section 8: HEALTH

Please tell me how much you agree or disagree with the following statements. You can answer: Agree a lot, agree somewhat, agree a little, neither agree nor disagree, disagree a little, disagree somewhat and disagree a lot.

	Agree a lot	Agree somewh at	Agree a little	Neither agree nor disagree	Disagree a little	Disagree somewh at	Disagree a lot	Do Not Know	Skip

8.1 I eat enough vegetables to stay healthy	◯	◯	◯	◯	◯	◯	◯	◯	◯
8.2 I eat enough fruit to stay healthy	◯	◯	◯	◯	◯	◯	◯	◯	◯
8.3 I eat healthily	◯	◯	◯	◯	◯	◯	◯	◯	◯
8.4 I would like to eat more fruits and vegetables in order to lose weight	◯	◯	◯	◯	◯	◯	◯	◯	◯
8.5 I would like to eat more fruits and vegetables in order to protect myself against cancer	◯	◯	◯	◯	◯	◯	◯	◯	◯
8.6 I would like to eat more fruits and vegetables in order to lower my blood pressure	◯	◯	◯	◯	◯	◯	◯	◯	◯
8.7 I would like to eat more fruits and vegetables to lower risk my risk of disease or illness	◯	◯	◯	◯	◯	◯	◯	◯	◯
8.8 I would like to eat more organic fruit than I do now	◯	◯	◯	◯	◯	◯	◯	◯	◯
8.9 I would like to eat more organic vegetables than I do now	◯	◯	◯	◯	◯	◯	◯	◯	◯

Section 9: Self-Efficacy

SELF-EFFICACY
9.1 I would like for you to answer on a scale of 1 to 10, with 1 being 'not at all important', and 10 being "very important.
Considering all of the things that require your time, attention and money, how important is it for you to find ways to buy and fix more fruits and vegetables for yourself and your family?

◯ 1 ◯ 2 ◯ 3 ◯ 4
◯ 5 ◯ 6 ◯ 7 ◯ 8
◯ 9 ◯ 10 ◯ don't know
◯ skip

SELF-EFFICACY

There are a number of different ways that people find time to buy, fix, and eat more fruits and vegetables. We would like to know how easy or hard you would find each of the following things to do.

On a scale of 1 to 10 with 1 being "VERY EASY" and 10 being "VERY HARD"

How easy or hard would it be for you to...

	1 Very easy	2	3	4	5	6	7	8	9	10 Very Hard	Don't know	Skip

9.2. ...buy more fruits and vegetables than you normally do the next time you shop?	◯	◯	◯	◯	◯	◯	◯	◯	◯	◯	◯	◯
9.3. ...use all of the fruits and vegetables that you buy before they go bad?	◯	◯	◯	◯	◯	◯	◯	◯	◯	◯	◯	◯
9.4. ...work more fruits and vegetables than you normally do into meals for yourself and your family?	◯	◯	◯	◯	◯	◯	◯	◯	◯	◯	◯	◯
9.5. ...work more fruits and vegetables than you normally do into snacks for yourself and your family?	◯	◯	◯	◯	◯	◯	◯	◯	◯	◯	◯	◯
9.6. ...cook vegetables in a way that is appealing to your family?	◯	◯	◯	◯	◯	◯	◯	◯	◯	◯	◯	◯
9.7. ...make up a vegetable dish with what you have on hand?	◯	◯	◯	◯	◯	◯	◯	◯	◯	◯	◯	◯
9.8. ...try vegetables that you have not eaten before?	◯	◯	◯	◯	◯	◯	◯	◯	◯	◯	◯	◯
9.9. ...prepare and cook new recipes?	◯	◯	◯	◯	◯	◯	◯	◯	◯	◯	◯	◯

Section 10: USDA FSS

These next questions are about the food eaten in your household in the last 12 months, since (current month) of last year and whether you were able to afford the food you need.

USDA FSS:

[IF ONE PERSON IN HOUSEHOLD, USE "I" IN PARENTHETICALS, OTHERWISE, USE "WE."]

Which of these statements best describes the food eaten in your household in the last 12 months:

-enough of the kinds of food (I/we) want to eat;
-enough, but not always the kinds of food (I/we) want;
-sometimes not enough to eat;

or,
-often not enough to eat?

◯ Enough of the kinds of food we want to eat
◯ Enough but not always the kinds of food we want
◯ Sometimes not enough to eat
◯ Often not enough to eat
◯ DK or Refused

USDA FSS<br><br>Household Stage 1: Questions HH2-HH4 (asked of all households; begin scale items).<br><br>[IF SINGLE ADULT IN HOUSEHOLD, USE "I," "MY," AND "YOU" IN PARENTHETICALS; OTHERWISE, USE "WE," "OUR," AND "YOUR HOUSEHOLD."]<br><br>HH2. Now I'm going to read you several statements that people have made about their food situation. For these statements, please tell me whether the statement was often true, sometimes true, or never true for (you/your household) in the last 12 months-that is, since last (name of current month).<br><br>The first statement is "(I/We) worried whether (my/our) food would run out before (I/we) got money to buy more." Was that often true, sometimes true, or never true for (you/your household) in the last 12 months?	◯ Often true<br>◯ Sometimes true<br>◯ Never true<br>◯ DK or Refused
USDA FSS<br><br>HH3. "The food that (I/we) bought just didn't last, and (I/we) didn't have money to get more." Was that often, sometimes, or never true for (you/your household) in the last 12 months?	◯ Often true<br>◯ Sometimes true<br>◯ Never true<br>◯ DK or Refused
USDA FSS<br><br>HH4. "(I/we) couldn't afford to eat balanced meals." Was that often, sometimes, or never true for (you/your household) in the last 12 months?	◯ Often true<br>◯ Sometimes true<br>◯ Never true<br>◯ DK or Refused
USDA FSS<br><br>AD1. In the last 12 months, since last (name of current month), did (you/you or other adults in your household) ever cut the size of your meals or skip meals because there wasn't enough money for food?	◯ Yes<br>◯ No (Skip AD1a)<br>◯ DK (Skip AD1a)
USDA FSS<br><br>AD1a. How often did this happen-almost every month, some months but not every month, or in only 1 or 2 months?	◯ Almost every month<br>◯ Some months but not every month<br>◯ Only 1 or 2 months<br>◯ DK
USDA FSS<br><br>AD2. In the last 12 months, did you ever eat less than you felt you should because there wasn't enough money for food?	◯ Yes<br>◯ No<br>◯ DK
USDA FSS<br><br>AD3. In the last 12 months, were you every hungry but didn't eat because there wasn't enough money for food?	◯ Yes<br>◯ No<br>◯ DK

USDA FSS<br><br>AD4. In the last 12 months, did you lose weight because there wasn't enough money for food?	◯ Yes<br>◯ No<br>◯ DK
USDA FSS<br><br>AD5. In the last 12 months, did (you/you or other adults in your household) ever not eat for a whole day because there wasn't enough money for food?	◯ Yes<br>◯ No (Skip AD5a)<br>◯ DK (Skip AD5a)
USDA FSS<br><br>AD5a. How often did this happen-almost every month, some months but not every month, or in only 1 or 2 months?	◯ Almost every month<br>◯ Some months but not every month<br>◯ Only 1 or 2 months<br>◯ DK
USDA FSS 1	__________________________________
USDA FSS 2	__________________________________
USDA FSS 3	__________________________________
USDA FSS 4	__________________________________
USDA FSS 5	__________________________________
USDA FSS 6	__________________________________
USDA FSS 7	__________________________________
USDA FSS 8	__________________________________
USDA FSS 9	__________________________________
USDA FSS 10	__________________________________
USDA FSS TOTAL SCORE (0-10)	__________________________________

SECTION 11: BRFSS Fruits and Vegetables

IF PHASE 1: These next questions are about the fruits and vegetables you ate or drank during the past 30 days. Please think about all forms of fruits and vegetables including cooked or raw, fresh, frozen or canned. Please think about all meals, snacks, and food consumed at home and away from home.

IF PHASE 2: These next questions are about the fruits and vegetables you ate or drank during the 30 days before a mobile market was brought to [site]. Please think about all forms of fruits and vegetables including cooked or raw, fresh, frozen or canned. Please think about all meals, snacks, and food consumed at home and away from home.

I will be asking how often you ate or drank each one: for example, once a day, twice a week, three times a month, and so forth.

INTERVIEWER NOTE: If respondent responds less than once per month, put "0" times per month. If respondent gives a number without a time frame, ask:

"Was that per day, week, or month?"

BRFFS
11.1 During the past month, how many times per day, week or month did you drink 100% PURE fruit juices? Do not include fruit-flavored drinks with added sugar or fruit juice you made at home and added sugar to. Only include 100% juice.

INTERVIEWER NOTE: Do not include fruit drinks with added sugar or other added sweeteners like Kool-aid, Hi-C, lemonade, cranberry cocktail, Tampico, Sunny Delight, Snapple, Fruitopia, Gatorade, Power-Ade, or yogurt drinks.

Do not include fruit juice drinks that provide 100% daily vitamin C but include added sugar.

Do not include vegetable juices such as tomato and V8 if respondent provides but include in "other vegetables" question 11.6.

DO include 100% pure juices including orange, mango, papaya, pineapple, apple, grape (white or red), or grapefruit. Only count cranberry juice if the respondent's perception is that it is 100% juice with no sugar or artificial sweetener added. 100% juice blends such as orange-pineapple, orange-tangerine, cranberry-grape are also acceptable as are fruit-vegetable 100% blends. 100% pure juice from concentrate (i.e., reconstituted) is counted.

- ◯ per day
- ◯ per week
- ◯ per month
- ◯ never
- ◯ don't know/ Not sure
- ◯ Refused/Skip

BRFFS
11.1 100% PURE fruit juices?

How many times? ______________________

BRFFS
11.2 During the past month, not counting juice, how many times per day, week, or month did your eat fruit? Count fresh, frozen, or canned fruit.

READ ONLY IF NECESSARY: "Your best guess is fine. Include apples, bananas, applesauce, oranges, grape fruit, fruit salad watermelon, cantaloupe or musk melon, papaya, lychees, star fruit, pomegranates, mangos, grapes, and berries such as blueberries and strawberries."

INTERVIEWER NOTE: Do not count fruit jam, jelly, or fuit preserves.
Do not include dried fruit in ready to eat cereals.

DO include dried raisins, cran-raisins if respondent tells you -- but due to their small serving size they are not included in the prompt.

DO include cut up fresh, froozen, or canned fruit added to yougurt, cereal, jello and other meal items.

INCLUDE culturally and geographically appropriate fruits that are not mentioned (e.g. genip, soursop sugar apple, figs, tamarind, bread fruit, sea grapes, carambola, longans, lychees, akee, rambutan, etc.).

- ◯ per day
- ◯ per week
- ◯ per month
- ◯ never
- ◯ don't know/ Not sure
- ◯ Refused/Skip

BRFFS
11.2 FRUIT

How many times?

____

BRFFS
11.3 During the past month, how many times per day, week, or month did you eat cooked or canned beans, such as refried, baked, black, garbanzo beans, beans in soup, soybeans, edamame, tofu or lentils.

Do NOT include long green beans.

Read only if necessary: "Include round or oval beans or peas such as navy, pinto, split peas, cow peas, hummus, lentils, soy beans and tofu.

Do NOT include long green beans such as string beans, broad or winged beans, or pole beans."

INTERVIEWER NOTE: Include soybeans also called edamame, TOFU (BEAN CURD MADE FROM SOYBEANS), kidney, pinto, hummus, lentils, black, black-eyed peas, cow peas, lima beans and white beans.

Include bean burgers including garden burgers and veggie burgers.

Include falafel and tempeh.

- ◯ per day
- ◯ per week
- ◯ per month
- ◯ never
- ◯ don't know/ Not sure
- ◯ Refused/Skip

BRFFS
11.3 BEANS

How many times?

____

BRFFS
11.4 During the past month, how many times per day, week, or month did you eat dark green vegetables for example broccoli or dark leafy greens including romaine, chard, collard greens or spinach?

INTERVIEWER NOTE: Each time a vegetable is eaten it counts as one time.

INTERVIEWER NOTE: Include all raw leafy green salads including spinach, mesclun, romaine lettuce, bok choy, dark green leafy lettuce, dandelions, komatsuna, watercress, and arugula.

Do not include iceberg (head) lettuce if specifically told type of lettuce. Include all cooked greens including kale, collard greens, choys, turnip greens, mustard greens.

- ◯ per day
- ◯ per week
- ◯ per month
- ◯ never
- ◯ don't know/ Not sure
- ◯ Refused/Skip

BRFFS
11.4 Dark Green Vegetables

How many times?

____

BRFFS
11.5 During the past month, how many times per day, week, or month did you eat orange-colored vegetables such as sweet potatoes, pumpkin, winter squash, or carrots?

Read only if needed: "Winter squash have hard, thick skins and deep yellow to orange flesh. They include acorn, buttercup, and spaghetti squash."
FOR INTERVIEWER: Include all forms of carrots including long or baby-cut.

Include carrot-slaw (e.g. shredded carrots with or without other vegetables or fruit).

Include all forms of sweet potatoes including baked, mashed, casserole, pie, or sweet potatoes fries.

Include all hard-winter squash varieties including acorn, autumn cup, banana, butternut, buttercup, delicate, hubbard, kabocha (Also known as an Ebisu, Delica, Hoka, Hokkaido, or Japanese Pumpkin; blue kuri), and spaghetti squash. Include all forms including soup.
Include pumpkin, including pumpkin soup and pie.

Do not include pumpkin bars, cake, bread or other grain-based desert-type food containing pumpkin (i.e. similar to banana bars, zucchini bars we do not include).

- ◯ per day
- ◯ per week
- ◯ per month
- ◯ never
- ◯ don't know/ Not sure
- ◯ Refused/Skip

BRFFS

Orange-Colored Vegetables

How many?

____

BRFFS
11.6 Not counting what you just told me about, during the past month, about how many times per day, week, or month did you eat OTHER vegetables? Examples of other vegetables include tomatoes, tomato juice or V-8 juice, corn, eggplant, peas, lettuce, cabbage, and white potatoes that are not fried such as baked or mashed potatoes.

Read only if needed: "Do not count vegetables you have already counted and do not include fried potatoes."

INTERVIEWER NOTE: Include corn, peas, tomatoes, okra, beets, cauliflower, bean sprouts, avocado, cucumber, onions, peppers (red, green, yellow, orange); all cabbage including American-style cole-slaw; mushrooms, snow peas, snap peas, broad beans, string, wax-, or pole-beans.
Include any form of the vegetable (raw, cooked, canned, or frozen).

Do include tomato juice if respondent did not count in fruit juice.

Include culturally and geographically appropriate vegetables that are not mentioned (e.g. daikon, jicama, oriental cucumber, etc.).

Do not include rice or other grains.

Do not include products consumed usually as condiments including ketchup, catsup, salsa, chutney, relish.

- ◯ per day
- ◯ per week
- ◯ per month
- ◯ never
- ◯ don't know/ Not sure
- ◯ Refused/Skip

BRFFS
OTHER VEGETABLES

How many?

____

---

unit * frequency

____

BRFFS unit * frequency

____

BRFFS unit*frequency

____

BRFFS unit * frequency

____

BRFFS unit * frequency

____

BRFSS unit * frequency

____

BRFSS: Juice times per month

____

BRFSS: Fruits per month

____

BRFSS: Beans per month

____

BRFSS: Dark Green Vegetables per month

____

BRFSS: Orange Colored Vegetables per Month

____

BRFSS: Other Vegetables per Month

____

BASELINE SURVEY WRAP-UP

At this time, we've finished with all of the questions from the Veggie Van Baseline/12-Month Survey.

Before we hang up, I'd like to confirm that none of your personal information, like address or email have not changed since the last time we spoke.

____

[event_1_arm_1][phone_number]
[event_1_arm_1][email_address]

[event_1_arm_1][mailing_1]
[event_2_arm_2][mailing_1]
[event_1_arm_1][mailing_2],
[event_1_arm_1][mailing_3] [event_1_arm_1][mailing_4]

[event_1_arm_1] [giftcard_prefer1]

NOTE TO INTERVIEWER: Update Participant Status after call!

We would like to thank you for your participation in the Veggie Van Study and for taking the time to complete this Survey. You should receive your $15 gift card for completing this survey within the next 1-3 weeks.

I also want to take this time to remind you that your next tasks will be 2 phone diet recalls. We will call you twice within the next two weeks to do those during the times that you said you would be available. One call will be on a weekday and the other will be on a weekend day. If you miss a call from us don't worry but please try to call us back; it's important that we do these diet recalls within a two-week time frame. You will receive a $15 gift card after you have completed both of those calls.

We thank you for your time. Have a great day!

We would like to thank you for your participation in the Veggie Van Study and for taking the time to complete this Survey. You should receive your $15 gift card for completing this survey within the next 1-3 weeks.

I also want to take this time to remind you that your next tasks will be 2 phone diet recalls and attending an in person data collection event at [site_name_1]. We will call you twice within the next two weeks to complete the diet recalls during the times that you said you would be available. One call will be on a weekday and the other will be on a weekend day. If you miss a call from us don't worry but please try to call us back; it's important that we do these diet recalls within a two week time frame. You will receive a $15 gift card after you have completed both those calls.

We thank you for your time. Have a great day!

We will be sending you a Food Amounts Booklet in the mail within the next few days. It will come in a manila envelope. You'll use it during the diet recalls so please keep it close-by.

We will be sending you a Food Amounts Booklet in the mail within the next few days. It will come in a manila envelope. You'll use it during the diet recalls so please keep it close-by.

Additionally, we will be holding an in person data collection event at [site_name_1] in partnership with [partner_name]. We will communicate details about the event in the near future. You can also reach out to [partner_name] or follow their social media for more details about the event. You will receive another $15 gift after completing the in person data collection.

Thank you again for participating, have a great day [baseline_data_coll_arm_1][first_name] [baseline_data_coll_arm_2][first_name] !

Did they complete the baseline survey?

- ◯ Baseline completed (Mark as Completed)
- ◯ Midway filled (Mark As Unverified)
- ◯ Not completed nor started (Mark Unverified)
- ◯ Other needs attention (Mark as Incomplete and write issue in the notes above)
