# Supplementary material for: Cluster-randomized controlled trial of a mobile produce market designed to address diet and food insecurity in underserved communities
Source: BMC Nutr. 2026 Apr 9;12:94. doi: 10.1186/s40795-026-01302-7 (PMC13182037; doi:10.1186/s40795-026-01302-7)
Supplement: Supplementary file 5 — Supplementary Material 5. Veggie Van Follow-Up Survey. [file 40795_2026_1302_MOESM5_ESM.pdf]

Veggie Van Follow-Up Survey

SECTION 1: Perceived Access

Please think about the places that you can buy groceries in the neighborhood near where you live. Think of your neighborhood as the area within a 20-minute walk or about one mile from your home. For each of the following statements, please tell me whether you strongly agree, agree, feel neutral, disagree, or strongly disagree.
[NOTE TO INTERVIEWER:] for questions in the following block, only read the response options if the participant cannot remember the answer choices

	Strongly Agree	Agree	Feel Neutral	Disagree	Strongly Disagree	Do Not Know	Skip

1.1 It is easy to buy fresh fruits and vegetables in my neighborhood.	○	○	○	○	○	○	○
1.2 There is a large selection of fruits and vegetables in my neighborhood.	○	○	○	○	○	○	○
1.3 The fresh fruits and vegetables in my neighborhood are high quality	○	○	○	○	○	○	○

PERCEIVED ACCESS
Please think about the places that you can buy groceries around [site_f_u]. Think of the area within a 20-minute walk or about one mile from [site_f_u]. For each of the following statements, please tell me whether you strongly agree, agree, feel neutral, disagree, or strongly disagree.
[NOTE TO INTERVIEWER:] for questions in the following block, only read the response options if the participant cannot remember the answer choices.

	Strongly Agree	Agree	Feel Neutral	Disagree	Strongly Disagree	Do Not Know	Skip
1.4 It is easy to buy fresh fruits and vegetables around [site_f_u].	○	○	○	○	○	○	○
1.5 There is a large selection of fruits and vegetables available around [site_f_u].	○	○	○	○	○	○	○
1.6 The fresh fruits and vegetables around [site_f_u] are high quality.	○	○	○	○	○	○	○

PERCEIVED ACCESS
Now, please think about your ability to find and purchase fresh fruits and vegetables in general. Again please tell me whether you strongly agree, agree, feel neutral, disagree, or strongly disagree.
[NOTE TO INTERVIEWER:] for questions in the following block, only read the response options if the participant cannot remember the answer choices

	Strongly Agree	Agree	Feel Neutral	Disagree	Strongly Disagree	Do Not Know	Skip
1.7 It is easy for me to get to a place where I can buy fresh fruits and vegetables.	○	○	○	○	○	○	○
1.8 I can afford to buy enough fresh fruits and vegetables for myself or my family.	○	○	○	○	○	○	○

1.9 It is easy for me to find a variety of fresh fruits and vegetables.	◯	◯	◯	◯	◯	◯	◯
1.10 It is easy for me to find high quality fresh fruits and vegetables.	◯	◯	◯	◯	◯	◯	◯

Compared to before the COVID-19 or Coronavirus pandemic, is it easier or harder to find fresh fruits and vegetables?

- ◯ It is about the same as before
- ◯ It has gotten easier to find fresh fruits and vegetables
- ◯ It has gotten harder to find fresh fruits and vegetables
- ◯ Don't know
- ◯ Skip

SECTION 2: Participant Demographics & Body Composition

The next group of questions will ask about you and your family. Just a reminder, any individual information you provide during this survey will be kept completely private. You are not required to answer any questions that you don't want to. If you would like to skip any question, just say "I choose not to answer this question, "and I will move on.

DEMOGRAPHICS
2.1 What is your gender?

Note to Interviewer: Do Not Read Choices

- ◯ Male
- ◯ Female
- ◯ Other
- ◯ Skip

DEMOGRAPHICS
2.2 What is your birthdate? (month - day - year) ________________________

BODYCOMP
2.3 How tall are you?
(inches) Note to interviewer: Do not read answer choices. If participant is having trouble answering, ask for his/her best guess. ________________________________

If participant gives you feet + inches, i.e. "I'm 5 foot 10" use following table:

4'6" = 54 inches
4'7" = 55 inches
4'8" = 56 inches
4'9" = 57 inches
4'10" = 58 inches
4'11" = 59 inches
5'0" = 60 inches
5'1" = 61 inches
5'2" = 62 inches
5'3' = 63 inches
5'4" = 64 inches
5'5" = 65 inches
5'6" = 66 inches
5'7" = 67 inches
5'8" = 68 inches
5'9" = 69 inches
5'10" = 70 inches
5'11" = 71 inches
6'0" = 72 inches
6'1" = 73 inches
6'2" = 74 inches
6'3" = 75 inches
6'4" = 76 inches
6'5" = 77 inches
6'6" = 78 inches
6'7" = 79 inches
6'8" = 80 inches
6'9" = 81 inches
6'10" = 82 inches
6'11" = 83 inches
7' 0" = 84 inches

BODYCOMP
2.4 How much do you weigh right now? (in POUNDS) ________________________________
[Note to interviewer: If participant is having trouble answering, ask for his/her best guess.]

BODYCOMP:
Height in centemeters (cm) ________________________________

BODYCOMP: Weight in kilograms (kg) ________________________________

BODYCOMP:
Body Mass Index (BMI) ________________________________

DEMOGRAPHICS<br>2.5 What is your current marital status?<br><br>[Note to interviewer:] Don't read answer choices.	◯ Married or living with a partner<br>◯ Single<br>◯ Divorced<br>◯ Separated<br>◯ Widowed<br>◯ Skip
DEMOGRAPHICS<br>2.6 What is the highest level of education you have completed?<br><br>[Note to Interviewer:] Don't read choices unless participant is having trouble.	◯ 8th Grade or less<br>◯ Some high school<br>◯ High School graduate or GED<br>◯ Trade or beauty school graduate<br>◯ Some College<br>◯ College graduate<br>◯ More than college (Some post graduate, post graduate, or professional degree<br>◯ Skip
DEMOGRAPHICS<br>2.7 Which of the following best describes your yearly household income?<br>[Note to Interviewer:] This question is asking for the yearly household income, not just the income of the participant.	◯ Lesss than $10,000<br>◯ $10,000 to $19,999<br>◯ $20,000 to $29,999<br>◯ $30,000 to $39,999<br>◯ $40,000 to $49,999<br>◯ $50,000 to $59,999<br>◯ $60,000 or more<br>◯ Skip
DEMOGRAPHICS<br>2.8 Do you consider yourself Hispanic or Latino/Latina?	◯ Yes- HISPANIC/LATINO/LATINA<br>◯ No<br>◯ Skip
DEMOGRAPHICS<br>2.9A What race(s) do you consider yourself? May select as many as applicable.<br><br>Note to Interviewer: Do not read answer choices.	☐ American Indian or Alaskan Native<br>☐ Asian<br>☐ Black/African American<br>☐ Native Hawaiian or Other Pacific Islander<br>☐ White<br>☐ Other (Specify)<br>☐ Skip
DEMOGRAPHICS<br>2.9A Specify what "Other Race" you consider yourself.	__________________________________
DEMOGRAPHICS<br>2.9B Among the following choices, what race do you most identify with? (single answer)	◯ American Indian or Alaskan Native<br>◯ Asian<br>◯ Black/African American<br>◯ Hispanic or Latino/Latina<br>◯ Native Hawaiian or Other Pacific Islander<br>◯ White<br>◯ Other (Specify)<br>◯ Skip
DEMOGRAPHICS<br>2.9B Specify what "Other Race" do you most identify with.	__________________________________

What is your current employment status?

- ◯ Working full-time
- ◯ Working part-time
- ◯ Retired
- ◯ SSI/Disability
- ◯ Not currently working
- ◯ Working a contract or temporary position
- ◯ Working a gig job (delivery, uber, etc.)
- ◯ Skip

Has your employment status changed since the COVID-19 or Coronavirus pandemic started? [select all that apply]

- ☐ Yes, I was laid off
- ☐ Yes, I quit my job
- ☐ Yes, I am working fewer hours
- ☐ Yes, I have been furloughed
- ☐ Yes, I have been required to work from home
- ☐ Yes, I am working more hours
- ☐ Yes, I changed jobs
- ☐ No, I have not had any job changes
- ☐ I don't work, not applicable

DEMOGRAPHICS
2.10 I'm going to list the names of some programs. Please tell me if you or anyone in your household has participated in each of the following programs within the past 12 months. [Note to Interviewer: check ALL that apply.]

Note to interviewer: Temporary Assistance for Needy Families or Welfare may be referred to as TANF or cash

- ☐ WIC
- ☐ SNAP (Food Stamps)
- ☐ Free or reduced-price school breakfast or lunch
- ☐ Head Start
- ☐ Food Pantry
- ☐ Medicaid
- ☐ Temporary Assistance for Needy Families or Welfare (or TANF or cash)
- ☐ Social Security Disability Benefits
- ☐ None
- ☐ Skip

Were you or anyone in your household eligible for Pandemic EBT or P-EBT benefits in the past year?

- ◯ Yes and we used it to purchase food for our household
- ◯ Yes, but we did not use it to purchase food for our household
- ◯ No
- ◯ Don't know
- ◯ Skip

Did you or anyone in your household receive boxes of food through the USDA Farm to Families Food Box which included distribution of free farm products which could have included F& V meat dairy program in the past year?
Note to interviewer: not food pantry

- ◯ Yes, We received 1-2 boxes
- ◯ Yes, We received 3-4 boxes
- ◯ Yes, we received 5 or more boxes
- ◯ No, we did not receive any boxes

Did you or anyone in your household receive unemployment in the past year?

- ◯ Yes
- ◯ No
- ◯ Don't know
- ◯ Skip

Have you moved from where you lived at the time of the baseline survey (about 12 months ago)?

Note to interviewer: if participant is unsure, say, "Your address at that time was [baseline_data_coll_arm_1][mailing_1] [baseline_data_coll_arm_2][mailing_1]."

- ◯ Yes
- ◯ No
- ◯ Don't know
- ◯ Skip

What is your current address?

____

DEMOGRAPHICS
2.11 How long have you resided at your current location? (IF more than 1 year, enter in whole years).

Note to Interviewer: ENTER data in YEARS.
For residency < 1 year:
1 month = .08 | 2 months = .16
3 months = .25 | 4 months = .33
5 months = .41 | 6 months = .5
7 months = .58 | 8 months = .66
9 months = .74 |10 months = .82
11 months = .90

____

DEMOGRAPHICS
2.12 How many adults for whom you buy food live in your household? Please include all adults 18 years of age or older, including yourself, in this number.

____

DEMOGRAPHICS
2.13 How many children for whom you buy food live in your household? Please include all children younger than 18 years of age in this number.

____

DEMOGRAPHICS
Mouths to feed total

____

Section 3 HDQ: Health and Diet Questions (partial)

The next questions are about your health.

3.1. In the past 12 months, has a health care provider told you that you have either of the following conditions?

	yes	no	don't know	skip
High blood pressure	◯	◯	◯	◯
Diabetes	◯	◯	◯	◯

HDQ
3.2 How do you manage your Diabetes (choose all that apply)

- ☐ diet
- ☐ oral medication
- ☐ insulin
- ☐ other

HDQ
3.2a. Please specify the "OTHER" methods you use to manage your diabetes.

____

HDQ
3.3 Are you currently following a special diet?

- ◯ yes
- ◯ no
- ◯ don't know/skip

HDQ<br>3.3a. What type of special diet?	☐ for diabetes<br>☐ low fat<br>☐ low salt<br>☐ weight loss<br>☐ vegetarian<br>☐ gluten free<br>☐ other (specify)<br>☐ don't know
HDQ<br>3.3b. What type of OTHER diet?	__________________________
HDQ<br>3.4. In general, would you say your health is:	○ Excellent<br>○ Very good<br>○ Good<br>○ Fair<br>○ Poor<br>○ Skip/Refused

SECTION 4: DIET MEASURES: FRUITS AND VEGETABLES

Now, I want you to think about what you usually ate in the last month. Please think about ALL the fruits and vegetables that you ate last month.

Include those that were:
raw and cooked.
eaten as snacks and as meals.
eaten at home and away from home (at restaurants, friends' houses, and/or take-out
and eaten alone and mixed with other foods

4.1 Thinking about ALL the vegetables you ate over the last month -- On average, how many servings of vegetables did you usually eat each day? Consider one serving of vegetables to be one medium-sized whole vegetable (about the size of a baseball), one cup of chopped or canned vegetables, or two cups of raw, leafy greens or lettuce.<br><br>[Note to Interviewer:] Enter the number of servings (in whole numbers) that the participant reports eating on the line provided. If the participant responds with "Less than one serving", enter 0.	○ Number of Servings<br>○ Do Not Know<br>○ Skip
3.2 Number of Vegetable Servings	__________________________
3.3 Thinking about ALL the fruits you ate over the past month -- on average, how many servings of fruit did you usually eat each day?<br><br>Consider one serving of fruit to be one medium-sized whole fruit (about the size of a baseball), or one cup of chopped or canned fruit.<br><br>Include only fresh, frozen, adn/or canned fruits, but not fruit juice.	○ Number of Servings<br>○ Do Not Know<br>○ Skip

4.4 Number of Fruit Servings

____

Compared to before the COVID-19 or Coronavirus pandemic, have you changed how often you eat fresh fruits and vegetables?

◯ No, it's about the same as before the pandemic
◯ Yes, I am eating more fresh fruits and vegetables
◯ No, I am eating fewer fresh fruits and vegetables

SECTION 5: BARRIERS

The next questions are about reasons that some people may find it easy or hard to eat enough fruits and vegetables. For each of the following statements, please tell me whether you strongly agree, agree, disagree, or strongly disagree.

Note to Interviewer:

For the following statements, only read each response options if the participant cannot remember the answer choices. If a statement does not apply to a participant (e.g. The participant does not work, and therefore cannot answer the statement about workplace availability , OR The participant lives alone and does not provide food for a family and therefore cannot answer the statement about the family's liking of fruits or vegetables), then choose "SKIP" for that statement.

	Strongly Agree	Agree	Disagree	Strongly Disagree	Do Not Know	Skip
1. It is easy for me to eat fruits and vegetables.	◯	◯	◯	◯	◯	◯
2. I do not have time to prepare fruits and vegetables.	◯	◯	◯	◯	◯	◯
3. I do not know how to prepare fruits and vegetables.	◯	◯	◯	◯	◯	◯
4. I do not have transportation to get to a place where I can get fruits and vegetables.	◯	◯	◯	◯	◯	◯
5. It cost too much money to buy fruits and vegetables.	◯	◯	◯	◯	◯	◯
6. I do not like fruits.	◯	◯	◯	◯	◯	◯
7. I do not like vegetables.	◯	◯	◯	◯	◯	◯
8. My family does not like fruits.	◯	◯	◯	◯	◯	◯
9. My familiy does not like vegetables	◯	◯	◯	◯	◯	◯
10. I do not have enough space to store fruits and vegetables in my home.	◯	◯	◯	◯	◯	◯

11. The restaurants I go to do not offer fruits and vegetables.	○	○	○	○	○	○
12. Fruits and vegetables are not available at my work (in the cafeteria or other vending outlets).	○	○	○	○	○	○
13. I cannot afford to buy organic vegetables	○	○	○	○	○	○
14. I cannot afford to buy organic fruit	○	○	○	○	○	○

Next, I am going to ask you about some ways that the Covid-19 pandemic may have made it easier or harder to buy fresh fruits and vegetables.

	Strongly Agree	Agree	Disagree	Strongly Disagree	Do Not Know	Skip
Fruits and vegetables have gotten more expensive since the pandemic started	○	○	○	○	○	○
I am not shopping as often and it is difficult to keep fresh fruits and vegetables on hand until the next time I shop	○	○	○	○	○	○
I'm concerned about the safety of public transportation, so I'm not shopping as much	○	○	○	○	○	○
I'm concerned that fresh fruits and vegetables are not safe to eat	○	○	○	○	○	○

BARRIERS:
reverse scoring for barriers item 1 ______________________

BARRIERS 1 scoring ______________________

BARRIERS 2 scoring ______________________

BARRIERS 3 scoring ______________________

BARRIERS 4 scoring ______________________

BARRIERS 5 scoring ______________________

BARRIERS 6 scoring ______________________

BARRIERS 7 scoring ______________________

BARRIERS 8 scoring ____________________

BARRIERS 9 scoring ____________________

BARRIERS 10 scoring ____________________

BARRIERS 11 scoring ____________________

BARRIERS 12 scoring ____________________

BARRIERS TOTAL SCORE ____________________

SECTION 6: SHOPPING

This next set of questions will ask you about shopping for food.

6.1 How Often do you usually shop for food?

Note to Interviewer: Don't read answer choices unless participant is having trouble answering.

- ◯ More than once a week
- ◯ Once a week
- ◯ Once every 1-2 weeks
- ◯ Once a month
- ◯ Less than once a month
- ◯ Do Not Know
- ◯ Skip

Compared to before the Coronavirus or COVID-19 pandemic, have you changed how often you usually shop for food?

- ◯ No, it's about the same as before the pandemic
- ◯ Yes, I am shopping more often
- ◯ Yes, I am shopping less often

In the past year, have you purchased groceries online?

If so, about how often?

- ◯ No
- ◯ Yes, a few times in the past year
- ◯ Yes, around once per month
- ◯ Yes, around once per week or almost once per week

Since the start of the coronavirus pandemic, have you changed how often you buy groceries online?

- ◯ No, it's about the same as before
- ◯ I am buying groceries online more often
- ◯ I am buying groceries online less often
- ◯ Other

Explain ____________________

6.2 At which type of store do you buy most of your food?<br>-----------------<br>[FOR INTERVIEWER:<br><br>Supermarket: Sells fresh meat (e.g., beef, pork, chicken, turkey) AND Has 4 or more cash registers AND Has at least two of the following service counters: Bakery, Butcher, or Deli<br><br>Grocery store: Sells fresh meat (e.g., beef, pork, chicken, turkey) AND Does not meet all the criteria for supermarket]. Examples of grocery stores include Trader Joe's, Aldi, Save A Lot and some ethnic and "mom-and-pop" food stores<br><br>Convenience Store - This includes small, independently-owned or chain stores that sell an edited selection of staple groceries and other convenience items, i.e., ready-to-heat and ready-to-eat foods. They often sell fresh milk and may have a deli or sell some processed meats (hot dogs, cold cuts, etc.) and other hot foods. Convenience stores are typically open long hours and some sell gasoline as well.]	◯ Supermarket or large grocery store ([region_f_u:value])<br>◯ Super-Center (Like Walmart or Target)<br>◯ Buying Club (like Sam's Club)<br>◯ Small grocery store<br>◯ Dollar store<br>◯ Corner store, gas station, or convenience store<br>◯ Farmers Market, farm stand, or CSA<br>◯ Mobile produce market<br>◯ Other (Specify below)<br>◯ Do Not Know<br>◯ Skip
At which type of store do you buy most of your food? Specify Other	________________________________
6.3 What are the names of the two places where you buy most of your food?<br><br>PLACE 1:	________________________________
6.3 Place 2	________________________________
6.4 Between [buystore1_f_u] and [buystore2_f_u] which store do you think you buy the most food from?	◯ Place 1 [buystore1_f_u]<br>◯ Place 2 [buystore2_f_u]
6.5 How do you usually shop for food from [buystore1_f_u]? Do you usually...	◯ Shop in person<br>◯ Shop online and have it delivered to your home<br>◯ Shop online and pick it up outside the store<br>◯ Other
Specify other method of shopping:	________________________________
6.5 How do you usually shop for food from [buystore2_f_u]? Do you usually...	◯ Shop in person<br>◯ Shop online and have it delivered to your home<br>◯ Shop online and pick it up outside the store<br>◯ Other
Specify other method of shopping:	________________________________

6.5 When you shop in person at [buymainstore_f_u], how do you usually travel there?	◯ Walk<br>◯ Bicycle<br>◯ Bus or other public transportation<br>◯ Drive a car or other motor vehicle.<br>◯ Get a ride with someone else<br>◯ Taxi<br>◯ Other (Please Specify)<br>◯ Do Not Know<br>◯ Skip
6.5 How do you usually travel to [buymainstore_f_u]? Specify Other mode of transport.	__________________________________
6.6 How long would it take you to get from your home to [buymainstore_f_u] if you walked there?<br><br>Note to interviewer: Don't read answer choices unless participant is having trouble.	◯ 10 minutes or less<br>◯ 11-20 minutes<br>◯ 21-30 minutes<br>◯ Do not know walk time, but I do know drive time (Specify)<br>◯ More than 30 minutes<br>◯ Do Not Know<br>◯ Skip
6.6 How long does it take to drive to [buymainstore_f_u]?	__________________________________
6.7 Where is [buymainstore_f_u]?<br><br>Please be as specific as you are able, giving the town, street, and/or nearby landmarks if possible.	__________________________________
6.8 At [buymainstore_f_u], how would you rate the price of fresh fruits and vegetables?	◯ Very affordable<br>◯ Somewhat affordable<br>◯ Somewhat expensive<br>◯ Very expensive<br>◯ Do Not Know<br>◯ Skip
6.9 At [buymainstore_f_u], how would you rate the quality of fresh fruits and vegetables?	◯ Very high quality<br>◯ Somewhat high quality<br>◯ Somewhat low quality<br>◯ Very low quality<br>◯ Do Not Know<br>◯ Skip
6.10 At [buymainstore_f_u] how would you rate the variety of fresh fruits and vegetables?	◯ Very high variety<br>◯ Somewhat high variety<br>◯ Somewhat low variety<br>◯ Very low variety<br>◯ Do Not Know<br>◯ Skip

6.11. On a scale of very important, somewhat important, a little important, not at all important:
How important are each of the following factors in your decision to shop at ([buymainstore_f_u])?

	Not at all important	A little important	Somewhat important	Very important	Do Not Know	Skip
a. Near your home	○	○	○	○	○	○
b. Near or on the way to other places where you spend time	○	○	○	○	○	○
c. Your friend/relatives shop at this store	○	○	○	○	○	○
d. Selection of foods	○	○	○	○	○	○
e. Quality of foods	○	○	○	○	○	○
f. Prices of foods	○	○	○	○	○	○
g. Access to public transportation	○	○	○	○	○	○
h. They offer an online shopping option	○	○	○	○	○	○

6.12a I'm going to read you a list of different types of stores. Please tell me how often you shop for fresh fruits and vegetables at each of the following types. When you answer, all you have to say is more than once a week, once a week, once every 1-2 weeks, once a month, rarely, or never.

For this section, only read answer choices if participant is having trouble answering.

(MAIN QUESTION)
How often do you buy fresh fruit and vegetables at...

	More than once a week	Once a week	Once every 1-2 weeks	Once a month	Rarely	Never	Do Not Know	Skip
...a supermarket (like [region_f_u:value])?	○	○	○	○	○	○	○	○
...a supercenter (like Walmart or Target)?	○	○	○	○	○	○	○	○
...a buying club (like Sam's Club, BJ's Warehouse, or Costco)	○	○	○	○	○	○	○	○
...a dollar store?	○	○	○	○	○	○	○	○
... a small grocery store?	○	○	○	○	○	○	○	○
...a corner store, gas station, or convenience store?	○	○	○	○	○	○	○	○

...a farmers market, farm stand, or CSA	◯	◯	◯	◯	◯	◯	◯	◯
...mobile produce market?	◯	◯	◯	◯	◯	◯	◯	◯
...and other type of store?	◯	◯	◯	◯	◯	◯	◯	◯

6.12b Please specify the other type of store that you shop at for fresh fruits and vegetables. ______________________________

6.13 What are the names of the two places where you buy most of your fresh fruits and vegetables? ______________________________

(Place 1)

6.13b {What are the names of the two places where you buy most of your fresh fruits and vegetables?} ______________________________

(Place 2)

SECTION 7: COOKING

COOKING
7.1 In a typical week, how many days do you eat a main meal that is prepared at home?

Notes to Interviewer: Do not read answer choices. If the participant's answer falls between two of the choices listed below, read the two answer choices to the participant and ask them to pick which is the most correct.

If needed say "A main meal is the most substantial or most filling meal of your day".

- ◯ Never -- I do not prepare any main meals at home
- ◯ 1-2 days per week
- ◯ 3-4 days per week
- ◯ 5-6 days per week
- ◯ Every day
- ◯ Do Not Know
- ◯ Skip

Compared to before the Coronavirus or COVID-19 pandemic, have you changed how often you usually cook at home?

- ◯ No, it is about the same as before the pandemic
- ◯ Yes, I am cooking at home more often
- ◯ Yes, I am cooking at home less often
- ◯ Other

Section 8: HEALTH

Please tell me how much you agree or disagree with the following statements. You can answer: Agree a lot, agree somewhat, agree a little, neither agree nor disagree, disagree a little, disagree somewhat and disagree a lot.

	Agree a lot	Agree somewhat	Agree a little	Neither agree nor disagree	Disagree a little	Disagree somewhat	Disagree a lot	Do Not Know	Skip
8.1 I eat enough vegetables to stay healthy	◯	◯	◯	◯	◯	◯	◯	◯	◯

8.2 I eat enough fruit to stay healthy	◯	◯	◯	◯	◯	◯	◯	◯	◯
8.3 I eat healthily	◯	◯	◯	◯	◯	◯	◯	◯	◯
8.4 I would like to eat more fruits and vegetables in order to lose weight	◯	◯	◯	◯	◯	◯	◯	◯	◯
8.5 I would like to eat more fruits and vegetables in order to protect myself against cancer	◯	◯	◯	◯	◯	◯	◯	◯	◯
8.6 I would like to eat more fruits and vegetables in order to lower my blood pressure	◯	◯	◯	◯	◯	◯	◯	◯	◯
8.7 I would like to eat more fruits and vegetables to lower risk my risk of disease or illness	◯	◯	◯	◯	◯	◯	◯	◯	◯
8.8 I would like to eat more organic fruit than I do now	◯	◯	◯	◯	◯	◯	◯	◯	◯
8.9 I would like to eat more organic vegetables than I do now	◯	◯	◯	◯	◯	◯	◯	◯	◯

Section 9: Self-Efficacy

SELF-EFFICACY
9.1 I would like for you to answer on a scale of 1 to 10, with 1 being 'not at all important', and 10 being "very important.
Considering all of the things that require your time, attention and money, how important is it for you to find ways to buy and fix more fruits and vegetables for yourself and you family?

◯ 1 ◯ 2 ◯ 3 ◯ 4
◯ 5 ◯ 6 ◯ 7 ◯ 8
◯ 9 ◯ 10 ◯ don't know
◯ skip

SELF-EFFICACY
There are a number of different ways that people find time to buy, fix, and eat more fruits and vegetables. We would like to know how easy or hard you would find each of the following things to do.

On a scale of 1 to 10 with 1 being "VERY EASY" and 10 being "VERY HARD"

How easy or hard would it be for you to...

	1 Very easy	2	3	4	5	6	7	8	9	10 Very Hard	Don't know	Skip
9.2. ...buy more fruits and vegetables than you normally do the next time you shop?	◯	◯	◯	◯	◯	◯	◯	◯	◯	◯	◯	◯

9.3. ...use all of the fruits and vegetables that you buy before they go bad?

9.4. ...work more fruits and vegetables than you nomrally do into meals for yourself and your family?

9.5. ...work more fruits and vegetables than you normally do into snacks for yourself and your family?

9.6. ...cook vegetables in a way that is appealing to your family?

9.7. ...make up a vegetable dish with what you have on hand?

9.8. ...try vegetables that you have not eaten before?

9.9. ...prepare and cook new recipes?

Section 10: USDA FSS

These next questions are about the food eaten in your household in the last 12 months, since (current month) of last year and whether you were able to afford the food you need.

USDA FSS:

[IF ONE PERSON IN HOUSEHOLD, USE "I" IN PARENTHETICALS, OTHERWISE, USE "WE."]

Which of these statements best describes the food eaten in your household in the last 12 months:

-enough of the kinds of food (I/we) want to eat;
-enough, but not always the kinds of food (I/we) want;
-sometimes not enough to eat;

or,
-often not enough to eat?

- Enough of the kinds of food we want to eat
- Enough but not always the kinds of food we want
- Sometimes not enough to eat
- Often not enough to eat
- DK or Refused

USDA FSS<br><br>Household Stage 1: Questions HH2-HH4 (asked of all households; begin scale items).<br><br>[IF SINGLE ADULT IN HOUSEHOLD, USE "I," "MY," AND "YOU" IN PARENTHETICALS; OTHERWISE, USE "WE," "OUR," AND "YOUR HOUSEHOLD."]<br><br>HH2. Now I'm going to read you several statements that people have made about their food situation. For these statements, please tell me whether the statement was often true, sometimes true, or never true for (you/your household) in the last 12 months-that is, since last (name of current month).<br><br>The first statement is "(I/We) worried whether (my/our) food would run out before (I/we) got money to buy more." Was that often true, sometimes true, or never true for (you/your household) in the last 12 months?	◯ Often true<br>◯ Sometimes true<br>◯ Never true<br>◯ DK or Refused
USDA FSS<br><br>HH3. "The food that (I/we) bought just didn't last, and (I/we) didn't have money to get more." Was that often, sometimes, or never true for (you/your household) in the last 12 months?	◯ Often true<br>◯ Sometimes true<br>◯ Never true<br>◯ DK or Refused
USDA FSS<br><br>HH4. "(I/we) couldn't afford to eat balanced meals." Was that often, sometimes, or never true for (you/your household) in the last 12 months?	◯ Often true<br>◯ Sometimes true<br>◯ Never true<br>◯ DK or Refused
USDA FSS<br><br>AD1. In the last 12 months, since last (name of current month), did (you/you or other adults in your household) ever cut the size of your meals or skip meals because there wasn't enough money for food?	◯ Yes<br>◯ No (Skip AD1a)<br>◯ DK (Skip AD1a)
USDA FSS<br><br>AD1a. How often did this happen-almost every month, some months but not every month, or in only 1 or 2 months?	◯ Almost every month<br>◯ Some months but not every month<br>◯ Only 1 or 2 months<br>◯ DK
USDA FSS<br><br>AD2. In the last 12 months, did you ever eat less than you felt you should because there wasn't enough money for food?	◯ Yes<br>◯ No<br>◯ DK
USDA FSS<br><br>AD3. In the last 12 months, were you every hungry but didn't eat because there wasn't enough money for food?	◯ Yes<br>◯ No<br>◯ DK

USDA FSS<br><br>AD4. In the last 12 months, did you lose weight because there wasn't enough money for food?	◯ Yes<br>◯ No<br>◯ DK
USDA FSS<br><br>AD5. In the last 12 months, did (you/you or other adults in your household) ever not eat for a whole day because there wasn't enough money for food?	◯ Yes<br>◯ No (Skip AD5a)<br>◯ DK (Skip AD5a)
USDA FSS<br><br>AD5a. How often did this happen-almost every month, some months but not every month, or in only 1 or 2 months?	◯ Almost every month<br>◯ Some months but not every month<br>◯ Only 1 or 2 months<br>◯ DK
USDA FSS 1	__________________________________
USDA FSS 2	__________________________________
USDA FSS 3	__________________________________
USDA FSS 4	__________________________________
USDA FSS 5	__________________________________
USDA FSS 6	__________________________________
USDA FSS 7	__________________________________
USDA FSS 8	__________________________________
USDA FSS 9	__________________________________
USDA FSS 10	__________________________________
USDA FSS TOTAL SCORE (0-10)	__________________________________

SECTION 11: BRFSS Fruits and Vegetables

These next questions are about the fruits and vegetables you ate or drank during the past 30 days. Please think about all forms of fruits and vegetables including cooked or raw, fresh, frozen or canned. Please think about all meals, snacks, and food consumed at home and away from home.

I will be asking how often you ate or drank each one: for example, once a day, twice a week, three times a month, and so forth.

INTERVIEWER NOTE: If respondent responds less than once per month, put "0" times per month. If respondent gives a number without a time frame, ask:

"Was that per day, week, or month?"

BRFFS
11.1 During the past month, how many times per day, week or month did you drink 100% PURE fruit juices? Do not include fruit-flavored drinks with added sugar or fruit juice you made at home and added sugar to. Only include 100% juice.

INTERVIEWER NOTE: Do not include fruit drinks with added sugar or other added sweeteners like Kool-aid, Hi-C, lemonade, cranberry cocktail, Tampico, Sunny Delight, Snapple, Fruitopia, Gatorade, Power-Ade, or yogurt drinks.

Do not include fruit juice drinks that provide 100% daily vitamin C but include added sugar.

Do not include vegetable juices such as tomato and V8 if respondent provides but include in "other vegetables" question 11.6.

DO include 100% pure juices including orange, mango, papaya, pineapple, apple, grape (white or red), or grapefruit. Only count cranberry juice if the respondent's perception is that it is 100% juice with no sugar or artificial sweetener added. 100% juice blends such as orange-pineapple, orange-tangerine, cranberry-grape are also acceptable as are fruit-vegetable 100% blends. 100% pure juice from concentrate (i.e., reconstituted) is counted.

- ◯ per day
- ◯ per week
- ◯ per month
- ◯ never
- ◯ don't know/ Not sure
- ◯ Refused/Skip

BRFFS
11.1 100% PURE fruit juices?

How many times?

____

BRFFS
11.2 During the past month, not counting juice, how many times per day, week, or month did your eat fruit? Count fresh, frozen, or canned fruit.

READ ONLY IF NECESSARY: "Your best guess is fine. Include apples, bananas, applesauce, oranges, grape fruit, fruit salad watermelon, cantaloupe or musk melon, papaya, lychees, star fruit, pomegranates, mangos, grapes, and berries such as blueberries and strawberries."

INTERVIEWER NOTE: Do not count fruit jam, jelly, or fuit preserves.
Do not include dried fruit in ready to eat cereals.

DO include dried raisins, cran-raisins if respondent tells you -- but due to their small serving size they are not included in the prompt.

DO include cut up fresh, froozen, or canned fruit added to yougurt, cereal, jello and other meal items.

INCLUDE culturally and geographically appropriate fruits that are not mentioned (e.g. genip, soursop sugar apple, figs, tamarind, bread fruit, sea grapes, carambola, longans, lychees, akee, rambutan, etc.).

- ◯ per day
- ◯ per week
- ◯ per month
- ◯ never
- ◯ don't know/ Not sure
- ◯ Refused/Skip

BRFFS
11.2 FRUIT

How many times?

____

BRFFS
11.3 During the past month, how many times per day, week, or month did you eat cooked or canned beans, such as refried, baked, black, garbanzo beans, beans in soup, soybeans, edamame, tofu or lentils.

Do NOT include long green beans.

Read only if necessary: "Include round or oval beans or peas such as navy, pinto, split peas, cow peas, hummus, lentils, soy beans and tofu.

Do NOT include long green beans such as string beans, broad or winged beans, or pole beans."

INTERVIEWER NOTE: Include soybeans also called edamame, TOFU (BEAN CURD MADE FROM SOYBEANS), kidney, pinto, hummus, lentils, black, black-eyed peas, cow peas, lima beans and white beans.

Include bean burgers including garden burgers and veggie burgers.

Include falafel and tempeh.

- ◯ per day
- ◯ per week
- ◯ per month
- ◯ never
- ◯ don't know/ Not sure
- ◯ Refused/Skip

BRFFS
11.3 BEANS

How many times?

____

BRFFS
11.4 During the past month, how many times per day, week, or month did you eat dark green vegetables for example broccoli or dark leafy greens including romaine, chard, collard greens or spinach?

INTERVIEWER NOTE: Each time a vegetable is eaten it counts as one time.

INTERVIEWER NOTE: Include all raw leafy green salads including spinach, mesclun, romaine lettuce, bok choy, dark green leafy lettuce, dandelions, komatsuna, watercress, and arugula.

Do not include iceberg (head) lettuce if specifically told type of lettuce. Include all cooked greens including kale, collard greens, choys, turnip greens, mustard greens.

- ◯ per day
- ◯ per week
- ◯ per month
- ◯ never
- ◯ don't know/ Not sure
- ◯ Refused/Skip

BRFFS
11.4 Dark Green Vegetables

How many times?

____

BRFFS
11.5 During the past month, how many times per day, week, or month did you eat orange-colored vegetables such as sweet potatoes, pumpkin, winter squash, or carrots?

Read only if needed: "Winter squash have hard, thick skins and deep yellow to orange flesh. They include acorn, buttercup, and spaghetti squash."
FOR INTERVIEWER: Include all forms of carrots including long or baby-cut.

Include carrot-slaw (e.g. shredded carrots with or without other vegetables or fruit).

Include all forms of sweet potatoes including baked, mashed, casserole, pie, or sweet potatoes fries.

Include all hard-winter squash varieties including acorn, autumn cup, banana, butternut, buttercup, delicate, hubbard, kabocha (Also known as an Ebisu, Delica, Hoka, Hokkaido, or Japanese Pumpkin; blue kuri), and spaghetti squash. Include all forms including soup.
Include pumpkin, including pumpkin soup and pie.

Do not include pumpkin bars, cake, bread or other grain-based desert-type food containing pumpkin (i.e. similar to banana bars, zucchini bars we do not include).

- ◯ per day
- ◯ per week
- ◯ per month
- ◯ never
- ◯ don't know/ Not sure
- ◯ Refused/Skip

BRFFS

Orange-Colored Vegetables

How many?

____

BRFFS
11.6 Not counting what you just told me about, during the past month, about how many times per day, week, or month did you eat OTHER vegetables? Examples of other vegetables include tomatoes, tomato juice or V-8 juice, corn, eggplant, peas, lettuce, cabbage, and white potatoes that are not fried such as baked or mashed potatoes.

Read only if needed: "Do not count vegetables you have already counted and do not include fried potatoes."

INTERVIEWER NOTE: Include corn, peas, tomatoes, okra, beets, cauliflower, bean sprouts, avocado, cucumber, onions, peppers (red, green, yellow, orange); all cabbage including American-style cole-slaw; mushrooms, snow peas, snap peas, broad beans, string, wax-, or pole-beans.
Include any form of the vegetable (raw, cooked, canned, or frozen).

Do include tomato juice if respondent did not count in fruit juice.

Include culturally and geographically appropriate vegetables that are not mentioned (e.g. daikon, jicama, oriental cucumber, etc.).

Do not include rice or other grains.

Do not include products consumed usually as condiments including ketchup, catsup, salsa, chutney, relish.

- ◯ per day
- ◯ per week
- ◯ per month
- ◯ never
- ◯ don't know/ Not sure
- ◯ Refused/Skip

BRFFS
OTHER VEGETABLES

How many? ________________________________

unit * frequency ________________________________

BRFFS unit * frequency ________________________________

BRFFS unit*frequency ________________________________

BRFFS unit * frequency ________________________________

BRFFS unit * frequency ________________________________

BRFSS unit * frequency ________________________________

BRFSS: Juice times per month ________________________________

BRFSS: Fruits per month

____

BRFSS: Beans per month

____

BRFSS: Dark Green Vegetables per month

____

BRFSS: Orange Colored Vegetables per Month

____

BRFSS: Other Vegetables per Month

____

Are you aware of a mobile produce market program at [site_f_u]?

◯ Yes
◯ No

Where is [site_f_u] in relation to your day-to-day activities?

◯ Near where I live
◯ Near where I work
◯ Near a place where I usually go (please specify)
◯ Other (please specify)

Please explain site relation-

____

Have you ever bought anything from the mobile market at [site_f_u]?
Note to Interviewer: Do not read answer choices.

◯ Yes
◯ No

Over the past month, how many times did you buy produce from the mobile market at [site_f_u]?
Note to Interviewer: Do not read answer choices.

◯ 0 times
◯ 1 time
◯ 2 times
◯ 3 times
◯ 4 times or more
◯ Do Not Know
◯ Skip

When you shop at the mobile market, how do you usually pay for your food?
Interviewer please review choices and select all that apply. Have you ever...

☐ Paid with Cash/Check
☐ Paid with Credit or Debit
☐ Paid with SNAP/EBT
☐ Paid with WIC EBT
☐ Used a WIC Farmers Market Nutrition Program Voucher
☐ Used a Senior Farmers Market Nutrition Program Voucher
☐ Used a SNAP matching program such as Double Up Food Bucks (note the program name if they know it)
☐ Used a Fruit and Veggie Prescription Voucher
☐ Used another type of produce incentive program (please specify)
☐ Received free donated produce at the market
☐ Other

Other payment method:

____

For this question, we are interested in whether the overall quantity of fruits and vegetables you purchased has changed, regardless of the price of the fruits and vegetables. Has shopping at the mobile market changed the amount of fruits and vegetables that you (or your family) buy(s)?	◯ YES -- I buy more fruits and vegetables.<br>◯ YES -- I buy fewer fruits and vegetables.<br>◯ NO -- I buy the same amount of fruits and vegetables as I did before buying from the mobile market.<br>◯ Do Not Know<br>◯ Skip
Has shopping at the mobile market changed the amount of fruits and vegetables that you (or your family) eat(s)?	◯ YES -- I eat more fruits and vegetables.<br>◯ YES -- I eat fewer fruits and vegetables.<br>◯ NO -- I eat the same amount of fruits and vegetables.<br>◯ Do Not Know<br>◯ Skip
Have you made any other changes because of shopping at the mobile market?<br>Note to Interviewer: Type participant's response as completely as possible into field provided. If participant does not provide any other types of changes, leave field blank.	________________________________
The next questions will ask for your opinion about the mobile market. While you are answering these questions, please remember that your individual responses will be kept private. After the completion of the research study, a summary of the findings from this survey will be used to help make the mobile market program better. Your personal responses will not be given to anyone who works at the mobile market.<br><br>How would you rate the price of fresh fruits and vegetables sold at the mobile market at [site_f_u]?	◯ Very affordable<br>◯ Somewhat affordable<br>◯ Somewhat expensive<br>◯ Very expensive<br>◯ Do Not Know<br>◯ Skip
How would you rate the quality of fresh fruits and vegetables sold at the mobile market at [site_f_u]?	◯ Very high quality<br>◯ Somewhat high quality<br>◯ Somewhat low quality<br>◯ Very low quality<br>◯ Do Not Know<br>◯ Skip
How would you rate the variety of fresh fruits and vegetables sold at the mobile market at [site_f_u]?	◯ Very high variety<br>◯ Somewhat high variety<br>◯ Somewhat low variety<br>◯ Very low variety<br>◯ Do Not Know<br>◯ Skip
We would like to know how important each of the following factors are in your decision to shop at the mobile market? On a scale of 1 to 5, with 1 being "not at all important"; and 5 being "very important"; -- How important is it that:<br><br>The mobile market is near your home?	◯ 1 - Not at all important<br>◯ 2<br>◯ 3<br>◯ 4<br>◯ 5 - Very Important<br>◯ Not Applicable<br>◯ Do Not Know<br>◯ Skip

The mobile market is near or on your way to other places where you usually go?	◯ 1 - Not at all important<br>◯ 2<br>◯ 3<br>◯ 4<br>◯ 5 - Very Important<br>◯ Not Applicable<br>◯ Do Not Know<br>◯ Skip
There is access to public transportation when traveling to the market?	◯ 1 - Not at all important<br>◯ 2<br>◯ 3<br>◯ 4<br>◯ 5 - Very Important<br>◯ Not Applicable<br>◯ Do Not Know<br>◯ Skip
Your friends or relatives shop at the mobile market?	◯ 1 - Not at all important<br>◯ 2<br>◯ 3<br>◯ 4<br>◯ 5 - Very Important<br>◯ Not Applicable<br>◯ Do Not Know<br>◯ Skip
How important is the variety of foods?	◯ 1 - Not at all important<br>◯ 2<br>◯ 3<br>◯ 4<br>◯ 5 - Very Important<br>◯ Not Applicable<br>◯ Do Not Know<br>◯ Skip
How important is the quality of foods?	◯ 1 - Not at all important<br>◯ 2<br>◯ 3<br>◯ 4<br>◯ 5 - Very Important<br>◯ Not Applicable<br>◯ Do Not Know<br>◯ Skip
How important are the prices of the food?	◯ 1 - Not at all important<br>◯ 2<br>◯ 3<br>◯ 4<br>◯ 5 - Very Important<br>◯ Not Applicable<br>◯ Do Not Know<br>◯ Skip

Please tell me how strongly you agree or disagree with the following two statements about the mobile market. You may respond with strongly agree, agree, feel neutral, disagree, or strongly disagree.<br><br>The mobile market is less expensive than other places I buy fruits and vegetables	◯ Strongly agree<br>◯ Agree<br>◯ Feel neutral<br>◯ Disagree<br>◯ Strongly disagree<br>◯ Do not know<br>◯ Skip
The mobile market sells the kinds of fruits and vegetables that I usually buy.	◯ Strongly agree<br>◯ Agree<br>◯ Feel neutral<br>◯ Disagree<br>◯ Strongly disagree<br>◯ Do not know<br>◯ Skip
When you shopped at the mobile market, do you recall any nutrition, cooking or food demonstrations or information?<br>Note to interviewer: do not read answers choices. Please check all that apply.	☐ Taste testing<br>☐ Cooking demonstrations<br>☐ Nutrition education<br>☐ Recipe cards<br>☐ Newsletter<br>☐ Other
You said you remember cooking demonstrations at the market. Please tell me how much you agree or disagree with the following statements about the cooking demos at the market. You can say that you strongly agree, agree, disagree, or strongly disagree.<br><br>The cooking demos helped me to try fruits and vegetables I don't usually buy	◯ Strongly agree<br>◯ Agree<br>◯ Feel neutral<br>◯ Disagree<br>◯ Strongly disagree<br>◯ Do not know<br>◯ Skip
The cooking demos helped me to use all of the produce I purchased at the market before it went bad.	◯ Strongly agree<br>◯ Agree<br>◯ Feel neutral<br>◯ Disagree<br>◯ Strongly disagree<br>◯ Do not know<br>◯ Skip
The cooking demos help me to prepare healthier meals and snacks.	◯ Strongly agree<br>◯ Agree<br>◯ Feel neutral<br>◯ Disagree<br>◯ Strongly disagree<br>◯ Do not know<br>◯ Skip
When you shopped at the mobile market, do you recall buying produce in a bundle or box? For example, you would purchase several produce items for one set price.	◯ Yes<br>◯ No
Please tell me how much you agree or disagree with the following statements about the produce bundles or boxes at the market. You can say that you strongly agree, agree, disagree, or strongly disagree.<br><br>The bundles helped me to try fruits and vegetables I don't usually buy.	◯ Strongly agree<br>◯ Agree<br>◯ Feel neutral<br>◯ Disagree<br>◯ Strongly disagree<br>◯ Do not know<br>◯ Skip

The bundles helped me to afford more fruits and vegetables than usual	◯ Strongly agree<br>◯ Agree<br>◯ Feel neutral<br>◯ Disagree<br>◯ Strongly disagree<br>◯ Do not know<br>◯ Skip
18. Do you know the name of the organization that runs the mobile market at [site_f_u]?<br>Note to interviewer: If they answer with the org name or the mobile market name then select yes.	◯ Yes<br>◯ No
19. Next, I want you to think about [city_org_f_u] that runs the mobile produce market at [site_f_u]. Please tell me how much you agree or disagree with each of the following statements.<br><br>I can influence the decisions made by [city_org_f_u] regarding their mobile market at [site_f_u].	◯ Strongly agree<br>◯ Agree<br>◯ Feel neutral<br>◯ Disagree<br>◯ Strongly disagree<br>◯ Do not know<br>◯ Skip
[city_org_f_u]'s mobile market at [site_f_u] has influence over decisions that affect my day-to-day life.	◯ Strongly agree<br>◯ Agree<br>◯ Feel neutral<br>◯ Disagree<br>◯ Strongly disagree<br>◯ Do not know<br>◯ Skip
[city_org_f_u] is effective in achieving its goals for [site_f_u]	◯ Strongly agree<br>◯ Agree<br>◯ Feel neutral<br>◯ Disagree<br>◯ Strongly disagree<br>◯ Do not know<br>◯ Skip
The [city_org_f_u]'s mobile market at [site_f_u] can influence decisions that affect the community.	◯ Strongly agree<br>◯ Agree<br>◯ Feel neutral<br>◯ Disagree<br>◯ Strongly disagree<br>◯ Do not know<br>◯ Skip
I am satisfied with the amount of influence I have over decisions that [city_org_f_u] makes about the mobile market at [site_f_u]	◯ Strongly agree<br>◯ Agree<br>◯ Feel neutral<br>◯ Disagree<br>◯ Strongly disagree<br>◯ Do not know<br>◯ Skip
Do you remember receiving any newsletters sent by [city_org_f_u] or [site_f_u]?	◯ Yes, I received newsletters when I went to the market<br>◯ Yes, I received newsletters by e-mail<br>◯ No

How often did you receive the newsletters?	◯ More than once a week<br>◯ Once a week<br>◯ 2-3 times per month<br>◯ Once a month<br>◯ Less than once a month<br>◯ Do Not Know<br>◯ Skip
How much of each newsletter did you read?	◯ None<br>◯ A little<br>◯ Some<br>◯ Most or all<br>◯ Do Not Know<br>◯ Skip
Please tell me how much you agree or disagree with the following statements about the newsletters you received. You can say that you strongly agree, agree, disagree, or strongly disagree.<br><br>The newsletters help to remind me to go to Mobile market.	◯ Strongly agree<br>◯ Agree<br>◯ Feel neutral<br>◯ Disagree<br>◯ Strongly disagree<br>◯ Do not know<br>◯ Skip
The newsletters help me to use all of the produce I received at Mobile market before it went bad.	◯ Strongly agree<br>◯ Agree<br>◯ Feel neutral<br>◯ Disagree<br>◯ Strongly disagree<br>◯ Do not know<br>◯ Skip
The newsletters help me to prepare healthier meals and snacks.	◯ Strongly agree<br>◯ Agree<br>◯ Feel neutral<br>◯ Disagree<br>◯ Strongly disagree<br>◯ Do not know<br>◯ Skip
What topics do you recall in the newsletters? (check all that apply)	☐ Market locations and times<br>☐ Tips on how to store and prepare the produce at the market<br>☐ Recipes<br>☐ Nutrition information<br>☐ Information about farmers or where the food came from<br>☐ Other (please specify)
Please specify	______________________

What were the main reasons that you did not shop at the mobile market?<br>Note to Interviewer: Do not read answer choices. Let the participant respond freely and mark all that apply.	☐ I do not know where the Mobile market is.<br>☐ I cannot use my Electronic Benefits Transfer (EBT) at the Mobile market.<br>☐ I cannot use WIC benefits at the Mobile market.<br>☐ I cannot use my credit or debit card at the Mobile market<br>☐ There is no public transportation to and from the Mobile market.<br>☐ I do not have my own car or transportation to and from the Mobile market.<br>☐ I do not know when the Mobile market is open.<br>☐ I do not know how to use or prepare the many foods sold at the Mobile market.<br>☐ The Mobile market hours do not fit with my schedule.<br>☐ The Mobile market is too expensive.<br>☐ The Mobile market does not let me choose the items in my box.<br>☐ I do not want to commit to a Mobile market subscription.<br>☐ Other<br>☐ Do Not Know<br>☐ Skip
How likely are you to use the Mobile market Program in the next month?	○ Very likely<br>○ Somewhat likely<br>○ Somewhat unlikely<br>○ Very unlikely<br>○ Do Not Know<br>○ Skip
Is there anything you would change about the Mobile market Program to make it better for you or other people in your community?<br>Note to Interviewer: Type as much as response from participant word-for-word as possible. If participant does not provide any feedback, says that they don't know, or skips/refuses to answer this question, leave the field blank.	______________________________
Are you aware of a mobile produce market program in your community?<br><br>Note to interviewer: If participant is unfamiliar with mobile markets, tell them that mobile produce markets, or mobile markets, are like mobile farmer's markets that travel and set up in different locations in communities to distribute and sell fruits, vegetables, and other food items. However, they are different than a Farmer's market in that food is usually sold by one organization rather than multiple famers.	○ Yes<br>○ No

Where is the Mobile Market program offered in your community?	◯ Veteran's One Stop<br>◯ Hispanics United<br>◯ Linden Park Early Childhood Education Center<br>◯ Bible Way<br>◯ Whitewater Middle School/Tuckaseegee Elementary School<br>◯ Grace Lutheran Church<br>◯ The WIRE<br>◯ Brigade Boys & Girls Club<br>◯ Grove Street Peoples Market<br>◯ Glouster Memorial Park<br>◯ Other
Please specify other	__________________________________
Where is [where_is_mm] in relation to your day-to-day activities?	☐ Near where I live<br>☐ Near where I work<br>☐ Near a place where I usually go (please specify)<br>☐ Other (please specify)
Please specify other	__________________________________
Have you ever bought anything from the mobile market at [where_is_mm]?<br><br>Note to Interviewer: Do not read answer choices.	◯ Yes<br>◯ No
Over the past month, how many times did you buy produce from the mobile market at [where_is_mm]?<br><br>Note to interview: Do not read answer choices	◯ 0 times<br>◯ 1 time<br>◯ 2 times<br>◯ 3 times<br>◯ 4 times or more<br>◯ Do Not Know<br>◯ Skip
When you shop at the mobile market at [where_is_mm], how do you usually pay for your food?<br><br>Note to interviewer: please review choices and select all that apply. Have you ever...	☐ Paid with Cash/Check<br>☐ Paid with Credit or Debit<br>☐ Paid with SNAP/EBT<br>☐ Paid with WIC EBT<br>☐ Used a WIC Farmers Market Nutrition Program Voucher<br>☐ Used a Senior Farmers Market Nutrition Program Voucher<br>☐ Used a SNAP matching program such as Double Up Food Bucks (note the program name if they know it)<br>☐ Used a Fruit and Veggie Prescription Voucher<br>☐ Used another type of produce incentive program (please specify)<br>☐ Received free donated produce at the market<br>☐ Other
Please specify	__________________________________

For this question, we are interested in whether the overall quantity of fruits and vegetables you purchased has changed, regardless of the cost of the fruits and vegetables. Has shopping at the mobile market at [where_is_mm] changed the amount of fruits and vegetables that you (or your family) buy(s)?	◯ YES -- I buy more fruits and vegetables.<br>◯ YES -- I buy fewer fruits and vegetables.<br>◯ NO -- I buy the same amount of fruits and vegetables as I did before buying from the mobile market.<br>◯ Do Not Know<br>◯ Skip
Has shopping at the mobile market at [where_is_mm] changed the amount of fruits and vegetables that you (or your family) eat(s)?	◯ YES -- I eat more fruits and vegetables.<br>◯ YES -- I eat fewer fruits and vegetables.<br>◯ NO -- I eat the same amount of fruits and vegetables.<br>◯ Do Not Know<br>◯ Skip
Have you made any other changes because of shopping at the mobile market?<br><br>Note to Interviewer: Type participant's response as completely as possible into field provided. If participant does not provide any other types of changes, leave field blank.	________________________________
The next questions will ask for your opinion about the mobile market at [where_is_mm]. While you are answering these questions, please remember that your individual responses will be kept private. After the completion of the research study, a summary of the findings from this survey will be used to help make the mobile market program better. Your personal responses will not be given to anyone who works at the mobile market.<br><br>How would you rate the price of fresh fruits and vegetables sold at [where_is_mm]?	◯ Very affordable<br>◯ Somewhat affordable<br>◯ Somewhat expensive<br>◯ Very expensive<br>◯ Do Not Know<br>◯ Skip
How would you rate the quality of fresh fruits and vegetables sold at [where_is_mm]?	◯ Very high quality<br>◯ Somewhat high quality<br>◯ Somewhat low quality<br>◯ Very low quality<br>◯ Do Not Know<br>◯ Skip
How would you rate the variety of fresh fruits and vegetables sold at [where_is_mm]?	◯ Very high variety<br>◯ Somewhat high variety<br>◯ Somewhat low variety<br>◯ Very low variety<br>◯ Do Not Know<br>◯ Skip
We would like to know how important each of the following factors are in your decision to shop at [where_is_mm]? On a scale of 1 to 5, with 1 being "not at all important"; and 5 being "very important"; -- How important is it that:<br><br>The mobile market is near your home?	◯ 1 - Not at all important<br>◯ 2<br>◯ 3<br>◯ 4<br>◯ 5 - Very important<br>◯ Not Applicable<br>◯ Do Not Know<br>◯ Skip

The mobile market is near or on the way to other places where you usually go?	◯ 1 - Not at all important<br>◯ 2<br>◯ 3<br>◯ 4<br>◯ 5 - Very important<br>◯ Not Applicable<br>◯ Do Not Know<br>◯ Skip
There is access to public transportation when traveling to the mobile market?	◯ 1 - Not at all important<br>◯ 2<br>◯ 3<br>◯ 4<br>◯ 5 - Very important<br>◯ Not Applicable<br>◯ Do Not Know<br>◯ Skip
Your friends or relatives shop at the mobile market at [where_is_mm]?	◯ 1 - Not at all important<br>◯ 2<br>◯ 3<br>◯ 4<br>◯ 5 - Very important<br>◯ Not Applicable<br>◯ Do Not Know<br>◯ Skip
How important is the variety of foods?	◯ 1 - Not at all important<br>◯ 2<br>◯ 3<br>◯ 4<br>◯ 5 - Very important<br>◯ Not Applicable<br>◯ Do Not Know<br>◯ Skip
How important is the quality of foods?	◯ 1 - Not at all important<br>◯ 2<br>◯ 3<br>◯ 4<br>◯ 5 - Very important<br>◯ Not Applicable<br>◯ Do Not Know<br>◯ Skip
How important is the prices of foods?	◯ 1 - Not at all important<br>◯ 2<br>◯ 3<br>◯ 4<br>◯ 5 - Very important<br>◯ Not Applicable<br>◯ Do Not Know<br>◯ Skip

Please tell me how strongly you agree or disagree with the following two statements about the mobile market at [where_is_mm]. You may respond with strongly agree, agree, feel neutral, disagree, or strongly disagree.

The mobile market at [where_is_mm] is less expensive than other places I buy fruits and vegetables.

- ◯ Strongly Agree
- ◯ Agree
- ◯ Feel Neutral
- ◯ Disagree
- ◯ Strongly Disagree
- ◯ Do Not Know
- ◯ Skip

The mobile market at [where_is_mm] sells the kinds of fruits and vegetables that I usually buy.

- ◯ Strongly Agree
- ◯ Agree
- ◯ Feel Neutral
- ◯ Disagree
- ◯ Strongly Disagree
- ◯ Do Not Know
- ◯ Skip

When you shopped at the mobile market at [where_is_mm], do you recall any nutrition, cooking or food demonstrations or information?

Please check all that apply.

Note to interviewer: Do not read answers choices.

- ☐ Taste testing
- ☐ Cooking demonstrations
- ☐ Nutrition education/lesson
- ☐ Recipe cards
- ☐ Newsletter
- ☐ Other

You said you remember cooking demonstrations at [where_is_mm]. Please tell me how much you agree or disagree with the following statements about the cooking demos at the market. You can say that you strongly agree, agree, disagree, or strongly disagree.

The cooking demos helped me to try fruits and vegetables I don't usually buy

- ◯ Strongly Agree
- ◯ Agree
- ◯ Disagree
- ◯ Strongly Disagree
- ◯ Do Not Know
- ◯ Skip

The cooking demos helped me to use all of the produce I purchased at the market before it went bad.

- ◯ Strongly Agree
- ◯ Agree
- ◯ Disagree
- ◯ Strongly Disagree
- ◯ Do Not Know
- ◯ Skip

The cooking demos help me to prepare healthier meals and snacks.

- ◯ Strongly Agree
- ◯ Agree
- ◯ Disagree
- ◯ Strongly Disagree
- ◯ Do Not Know
- ◯ Skip

When you shopped at [where_is_mm], do you recall buying produce in a bundle or box? For example, you would purchase several items of produce for one set price.

- ◯ Yes
- ◯ No

Please tell me how much you agree or disagree with the following statements about the produce bundles or boxes at [where_is_mm]. You can say that you strongly agree, agree, disagree, or strongly disagree.<br><br>The bundles helped me to try fruits and vegetables I don't usually buy.	◯ Strongly Agree<br>◯ Agree<br>◯ Disagree<br>◯ Strongly Disagree<br>◯ do Not Know<br>◯ Skip
The bundles helped me to afford more fruits and vegetables than usual	◯ Strongly Agree<br>◯ Agree<br>◯ Disagree<br>◯ Strongly Disagree<br>◯ do Not Know<br>◯ Skip
Do you know the name of the organization that runs the mobile market at [where_is_mm]?<br><br>Note to interviewer: The answer should be [city_org_f_u]. If the participant hasn't heard of that organization, then you can mark the answer to this question as "no"	◯ Yes<br>◯ No
Please specify name of organization	________________________________
Next, I want you to think about [city_org_f_u], which runs the mobile market at [site_f_u]. Please tell me how much you agree or disagree with each of the following statements:<br><br>I can influence the decisions made by [city_org_f_u].	◯ Strongly Agree<br>◯ Agree<br>◯ Disagree<br>◯ Strongly Disagree<br>◯ Do Not Know<br>◯ Skip
[city_org_f_u]'s mobile market at [site_f_u] has influence over decisions that affect my day-to-day life.	◯ Strongly Agree<br>◯ Agree<br>◯ Disagree<br>◯ Strongly Disagree<br>◯ Do Not Know<br>◯ Skip
[city_org_f_u] is effective in achieving its goals for their mobile market at [site_f_u]	◯ Strongly Agree<br>◯ Agree<br>◯ Disagree<br>◯ Strongly Disagree<br>◯ Do Not Know<br>◯ Skip
[city_org_f_u]'s mobile market at [site_f_u] can influence decisions that affect the community.	◯ Strongly Agree<br>◯ Agree<br>◯ Disagree<br>◯ Strongly Disagree<br>◯ Do Not Know<br>◯ Skip

I am satisfied with the amount of influence I have over decisions that [city_org_f_u] makes about the mobile market at [site_f_u].	◯ Strongly Agree<br>◯ Agree<br>◯ Disagree<br>◯ Strongly Disagree<br>◯ Do Not Know<br>◯ Skip
Do you remember receiving any newsletters sent by the [city_org_f_u] or [site_f_u]?	◯ Yes, I received newsletters when I went to the market<br>◯ Yes, I received newsletters by email<br>◯ No
How often did you receive the newsletters?	◯ More than once a week<br>◯ Once a week<br>◯ 2-3 times per month<br>◯ Once a month<br>◯ Less than once a month<br>◯ Do Not Know<br>◯ Skip
How much of each newsletter did you read?	◯ None<br>◯ A little<br>◯ Some<br>◯ Most or All<br>◯ Do Not Know<br>◯ Skip
Please tell me how much you agree or disagree with the following statements about the newsletters you received. You can say that you strongly agree, agree, disagree, or strongly disagree.<br><br>The newsletters help to remind me to go to mobile market at [site_f_u].	◯ Strongly Agree<br>◯ Agree<br>◯ Disagree<br>◯ Strongly Disagree<br>◯ Do Not Know<br>◯ Skip
The newsletters help me to use all of the produce I receive at the mobile market at [site_f_u] before it goes bad.	◯ Strongly Agree<br>◯ Agree<br>◯ Disagree<br>◯ Strongly Disagree<br>◯ Do Not Know<br>◯ Skip
The newsletters help me to prepare healthier meals and snacks.	◯ Strongly Agree<br>◯ Agree<br>◯ Disagree<br>◯ Strongly Disagree<br>◯ Do Not Know<br>◯ Skip
What topics do you recall being covered in the newsletters? (Check all that apply)	☐ Market locations and times<br>☐ Tips on how to store and prepare the produce at the market<br>☐ Recipes<br>☐ Nutrition information<br>☐ Information about farmers or where the food came from<br>☐ Other (please specify)

Please specify other

____

Are you aware of any activities to help plan for starting a mobile market at [site_f_u]? Examples of planning activities could include focus groups, meetings or surveys to get your feedback on what the market should sell, when it should happen, etc.

- ◯ Yes
- ◯ No

Over the past year, how many planning activities did you participate in?

- ◯ 0 times
- ◯ 1 time
- ◯ 2 times
- ◯ 3 times
- ◯ 4 times or more
- ◯ Do Not Know
- ◯ Skip

What types of planning activities for the mobile market did you take part in?

Note to interviewer: do not need to read choices. Check all that apply.

- ☐ Interest form to say I wanted a market at this location
- ☐ Focus group
- ☐ Community meeting
- ☐ Survey
- ☐ Other (please specify)

Please specify other

____

Where is the site of the mobile market you shop at in relation to your day-to-day activities?

- ☐ Near where I live
- ☐ Near where I work
- ☐ Near a place where I usually go
- ☐ Other (please specify below)

Please specify other

____

How likely are you to use a mobile market if it came to [site_f_u]?

- ◯ Very likely
- ◯ Somewhat likely
- ◯ Somewhat unlikely
- ◯ Very unlikely
- ◯ Do Not Know
- ◯ Skip

What would make you more likely to use the mobile market if it came to [site_f_u]?

____

What were the main reasons that you did not shop at that mobile market at [where_is_mm]?<br><br>Note to Interviewer: Do not read answer choices. Let the participant respond freely and mark all that apply.	☐ I do not know where the Mobile market is.<br>☐ I cannot use my Electronic Benefits Transfer (EBT) at the Mobile market.<br>☐ I cannot use WIC benefits at the Mobile market.<br>☐ I cannot use my credit or debit card at the Mobile market.<br>☐ There is no public transportation to and from the Mobile market.<br>☐ I do not have my own car or transportation to and from the Mobile market.<br>☐ I do not know when the Mobile market is open.<br>☐ I do not know how to use or prepare the many foods sold at the Mobile market.<br>☐ The Mobile market hours do not fit with my schedule.<br>☐ The Mobile market is too expensive.<br>☐ The Mobile market does not let me choose the items in my box.<br>☐ I do not want to commit to a Mobile market subscription.<br>☐ Other (please specify)<br>☐ Do Not Know<br>☐ Skip
Please specify other	__________________________________
How likely are you to use the mobile market program at [where_is_mm] in the next month?	◯ Very likely<br>◯ Somewhat likely<br>◯ Somewhat unlikely<br>◯ Very unlikely<br>◯ Do Not Know<br>◯ Skip
Is there anything you would change about the Mobile market program at [where_is_mm] to make it better for you or other people in your community?<br><br>Note to Interviewer: Type as much as response from participant word-for-word as possible. If participant does not provide any feedback, says that they don't know, or skips/refuses to answer this question, leave the field blank.	__________________________________
Are you aware of any activities to help plan for starting a mobile market at [site_f_u]?<br><br>Examples of planning activities could include focus groups, meetings or surveys to get your feedback on what the market should sell, when it should happen, etc.	◯ Yes<br>◯ No
Over the past year, how many planning activities did you participate in?	◯ 0 times<br>◯ 1 time<br>◯ 2 times<br>◯ 3 times<br>◯ 4 times or more<br>◯ Do Not Know<br>◯ Skip

What types of planning activities for the mobile market did you take part in?<br><br>Note to interviewer: do not need to read choices. Check all that apply.	☐ Interest form to say I wanted a market at this location<br>☐ Focus group<br>☐ Community meeting<br>☐ Survey,<br>☐ Other (please specify below)
Please specify other	__________________________________
Next, I want you to think about [city_org_f_u] which is planning to start a mobile market at [site_f_u]. Please tell me how much you agree or disagree with each of the following statements.<br><br>I can influence the decisions that the [city_org_f_u] makes about their mobile market at [site_f_u]	○ Strongly Agree<br>○ Agree<br>○ Disagree<br>○ Strongly Disagree<br>○ Do Not Know<br>○ Skip
[city_org_f_u] has influence over decisions that affect my day-to-day life	○ Strongly Agree<br>○ Agree<br>○ Disagree<br>○ Strongly Disagree<br>○ Do Not Know<br>○ Skip
[city_org_f_u] is effective in achieving its goals for the mobile market	○ Strongly Agree<br>○ Agree<br>○ Disagree<br>○ Strongly Disagree<br>○ Do Not Know<br>○ Skip
[city_org_f_u] can influence decisions that affect the community	○ Strongly Agree<br>○ Agree<br>○ Disagree<br>○ Strongly Disagree<br>○ Do Not Know<br>○ Skip
I am satisfied with the amount of influence I have over decisions that the [city_org_f_u] makes about the mobile market	○ Strongly Agree<br>○ Agree<br>○ Disagree<br>○ Strongly Disagree<br>○ Do Not Know,<br>○ Skip
Where is [site_f_u] in relation to your day-to-day activities?	☐ Near where I live<br>☐ Near where I work<br>☐ Near a place where I usually go<br>☐ Other (please specify below)
Please specify other	__________________________________

How likely are you to use a mobile market if it came to [site_f_u]?

- ◯ Very likely
- ◯ Somewhat likely
- ◯ Somewhat unlikely
- ◯ Very unlikely
- ◯ Do Not Know
- ◯ Skip

What would make you more likely to use the mobile market if it came to [site_f_u]?

____

SURVEY WRAP-UP

At this time, we've finished with all of the questions from the Veggie Van 12-Month Survey.

Before we hang up, I'd like to confirm that none of your personal information, like address or email have not changed since the last time we spoke.

____

[event_1_arm_1][phone_number]
[baseline_data_coll_arm_2][phone_number]
[event_1_arm_1][email_address]
[baseline_data_coll_arm_2][email_address]

[event_1_arm_1][mailing_1]
[baseline_data_coll_arm_2][mailing_1]
[event_1_arm_1][mailing_2],
[event_1_arm_1][mailing_3] [event_1_arm_1][mailing_4]
[baseline_data_coll_arm_2][mailing_2],
[baseline_data_coll_arm_2][mailing_3]
[baseline_data_coll_arm_2][mailing_4]

[baseline_data_coll_arm_1][giftcard_prefer1]
[baseline_data_coll_arm_2][giftcard_prefer1]

NOTE TO INTERVIEWER: Update Participant Status after call!

We would like to thank you for your participation in the Veggie Van Study and for taking the time to complete this Survey. You should receive your $15 gift card for completing this survey within the next 1-3 weeks.

I also want to take this time to remind you that your next tasks will be 2 phone diet recalls. We will call you twice within the next two weeks to do those during the times that you said you would be available. One call will be on a weekday and the other will be on a weekend day. If you miss a call from us don't worry but please try to call us back; it's important that we do these diet recalls within a two week time frame. You will receive a $15 gift card after each phone diet recall that you complete within the next 2 weeks.

Have you receive a Food Amounts Booklets in the mail from us?

[yes] Great, keep that close by-you will need that in the upcoming days to complete the diet recalls.

[no/ lost] (confirm mailing address) "it should be arriving soon in a manila envelope, keep that close by you will need it for the next task' (UPDATE: Participant Status that they need a FAB)

NOTE TO INTERVIEWER: Update Participant Status after call!

We will be sending you a Food Amounts Booklet in the mail within the next few days. It will come in a manila envelope. You'll use it during the diet recalls so please keep it close-by.
